# HPLC-ESI-MS^n^ Identification and NMR Characterization of Glucosyloxybenzyl 2*R*-Benzylmalate Deriva-Tives from *Arundina Graminifolia* and Their Anti-Liver Fibrotic Effects In Vitro

**DOI:** 10.3390/molecules24030525

**Published:** 2019-01-31

**Authors:** Qingqing Liu, Feiyi Sun, Yulin Deng, Rongji Dai, Fang Lv

**Affiliations:** Beijing Key Labrotary for Separation and Analysis in Biomedicine and Pharmaceuticals, School of Life Science, Beijing Institute of Technology, Beijing 100081, China; lqq765946474@163.com (Q.L.); sfyjet@163.com (F.S.); deng@bit.edu.cn (Y.D.); dairongji@bit.edu.cn (R.D.)

**Keywords:** *Arundina graminifolia*, glucosyloxybenzyl 2*R*-benzylmalates, MS^n^ fragmentation pattern, anti-liver fibrotic effects

## Abstract

Four new glucosyloxybenzyl 2*R*-benzylmalate derivatives, named Arundinoside H (**2**), I (**5**), J (**6**), K (**8**) as well as four known compounds Arundinoside D (**1**), G (**3**), F (**4**), E (**7**) were isolated and characterized by a combination of chemical and spectroscopic methods, including HR-ESI-MS, 1D and 2D NMR experiments. Besides, 24 unreported compounds were inferred from ESI-MS^n^ data. The anti-liver fibrotic activities of the isolates were determined as proliferation inhibition of lipopolysaccharide (LPS)-induced activation of rat hepatic stellate cells (HSC-T6). The result suggested Arundinosides D, H, F, I and K showed moderate inhibitory effects in vitro.

## 1. Introduction

*Arundina graminifolia* (D. Don) Hochr., a species widely distributed in subtropical Asia and known as bai-yang-jie in Chinese, has a long history of use as one of the major drugs in a formula “BaoGan Capusle” with the efficacy of heat clearing and detoxifying, dispersing blood and relieving pain, reducing inflammation and promoting urination and so on [1]. Previous phytochemical investigation focusing on the chloroform and ethyl acetate exacts of *A. graminifolia* had resulted in the separation of stilbenoids [2,3,4], phenols [5,6,7], flavonoids [8,9] and other ketones [3,10,11]. However, the works on the polar parts of the plant are few. 

In the course of our studies on pharmacology, it was proved that the formula “BaoGan Capusle” was effective in the treatment of hepatic fibrosis and liver injury of model rat [12,13,14,15]. As a continuing study on bioactive constituents of *A. graminifolia*, a series of phytochemical and biological experiments of the *n*-butanol (*n*-BuOH) extract was thus performed to yield the isolation of four new and four known glucosyloxybenzyl 2*R*-benzylmalates. In this paper, we described the isolation and structural elucidation of these derivatives, as well as their anti-liver fibrotic activities in vitro. Furthermore, the fragmentation pathways of eight isolates were studied in positive ESI-MS^n^, and then 24 unreported glucosyloxybenzyl 2*R*-benzylmalate derivatives were predicted by HPLC-ESI-MS^n^. 

## 2. Results and Discussion

**Figure 1:**
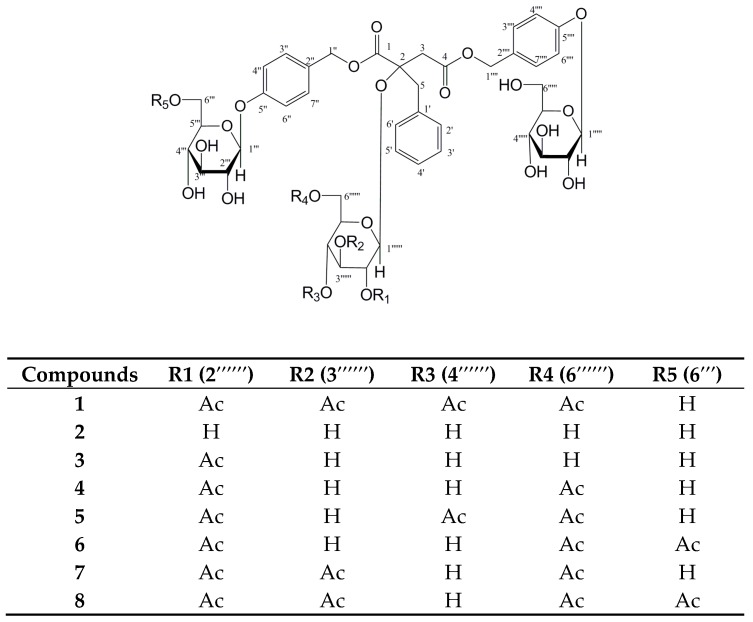
Structures of compounds **1**~**8.**

Through the combination of various chromatographic analyses, the *n*-BuOH extraction of *A. graminifolia* was separated carefully. Four new glucosyloxybenzyl 2*R*-benzylmalates Arundinoside H (**2**), I (**5**), J (**6**), K (**8**), as well as four known compounds Arundinoside D (**1**), G (**3**), F (**4**), E (**7**) [16] were obtained and determined by 1D and 2D NMR, and HR-ESI-MS spectra (see Supplementary Material). All these compounds were obtained as white amorphous powder. The ^1^H and ^13^C NMR data of the isolates were listed in Table 1 and Table 2, and their structures were shown in Figure 1. The target glucosyloxybenzyl 2*R*-benzylmalates in Table 4 were observed in the positive ion mode spectra (see Supplementary Material). 

### 2.1. Structure Elucidation of New Compounds

The HR-ESI-MS showed a [M + NH_4_]^+^ ion at *m*/*z* 1066.3764, from which the molecular formula of compound **6** was determined to be C_49_H_60_O_25_. The ^1^H and ^13^C NMR data (Table 1 and Table 2) showed signals for four methylene groups at δ_C_ 41.0 (C-3), δ_H_ 2.96 (1H, d, *J* = 17.8 Hz, H-3), 2.90 (1H, d, *J* = 17.8 Hz, H-3); δ_C_ 43.8 (C-5), δ_H_ 3.10 (1H, d, *J* = 13.8 Hz, H-5), 3.02 (1H, d, *J* = 14 Hz, H-5); δ_C_ 66.6 (C-1″), δ_H_ 5.00 (1H, d, *J* = 12 Hz, H-1″), 4.93 (1H, d, *J* = 12 Hz, H-1″); δ_C_ 66.2 (C-1′′′′), δ_H_ 5.00 (1H, d, *J* = 12 Hz, H-1′′′′), 4.99 (1H, d, *J* = 12 Hz, H-1′′′′). One quaternary carbon at δ_C_ 81.0 (C-2) and two carbonyl groups at δ_C_ 170.5 (C-1), and δ_C_ 170.1 (C-4) were ascertained by comparing ^13^C NMR and DEPT spectra, which indicated the basic structure as malic acid [17]. The HMBC correlarions from H_2_-3 to C-1, C-2 and C-4; H_2_-5 to C-1 and C-2, combined the comparison of 1D NMR spectra of compound 6 with those of Arundinoside D~F, indicated the presence of 2*R*-malic acid moiety.

Through the proton signals at δ_H_ 7.18 (2H, H-2′/6′), 7.01 (2H, H-3′/5′), 7.17 (1H, H-4′), and the ^13^C signals at δ_C_ 135.5 (C-1′), 128.3 (C-2′/6′), 130.9 (C-3′/5′), 127.1 (C-4′), the benzene group was identified, combining at C-5 based on HMBC correlation between C-1′ and H_2_-5. Other two benzene groups were identified by the proton signals at δ_H_ 7.01 (4H, H-4″/6″/H-4′′′′/6′′′′), 7.23 (2H, H-3′′′′/7′′′′), 7.27 (2H, H-3″/7″), and the carbon signals at δ_C_ 157.9 (C-5″), 157.6 (C-5′′′′), 130.3 (C-3″/7″/C-3′′′′/7′′′′), 129.3 (C-2″/2′′′′), 116.7 (C-4″/6″), 116.5 (C-4′′′′/6′′′′). According to HMBC correlations between H_2_-1″ and C-2″, H_2_-1″″ and C-2′′′′, the substitution positions of the benzene groups were C-1″ and C-1′′′′, respectively.

The ^1^H NMR spectrum of compound **6** showed well-resolved signals for three anomeric protons of three glucoses at δ_H_ 4.92 (2H, d, *J* = 7.5 Hz, H-Glc-1′′′/1′′′′′) and 4.86 (1H, d, *J* = 7.5 Hz, H-Glc-1′′′′′′). The splitting patterns of anomeric proton signals indicated that the sugar units were β-linkage [18]. The long-correlations from H-1′′′ to C-5″, H-1′′′′′ to C-5′′′′, H-1′′′′′′ to C-2 in HMBC experiment ascertained the sugar units combined at C-5″, C-5′′′′ and C-2, respectively. The absolute configuration of the glucoses was d-form by the hydrolysis process [19].

**Figure 2:**
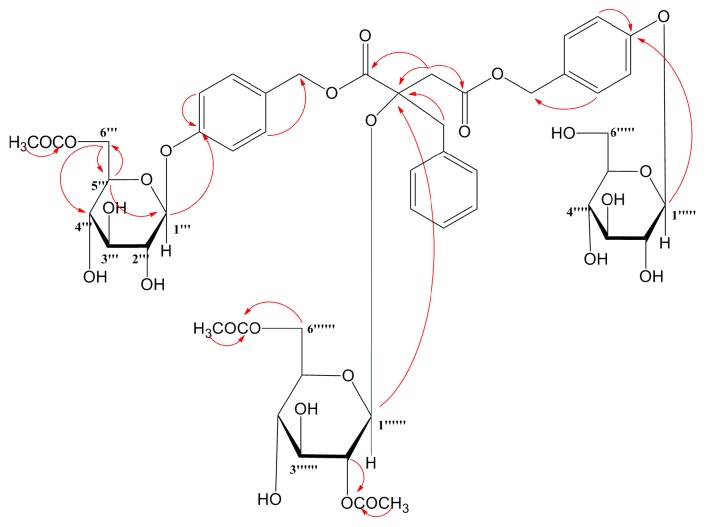
Key HMBC correlations of compound **6.**

In ^1^H and ^13^C NMR spectra, acetyl methyl protons at δ_H_ 1.72 (s), 1.92 (s), 1.99 (s) and acetyl carbonyl carbons at δ_C_ 169.8 (C), 170.7 (2C) indicated compound **6** possessed three acetyl groups, and the substitution positions were C-6′′′, C-2′′′′′′, C-6′′′′′′ by HMBC correlations from δ_H_ 4.27/4.08 (2H, m, H_2_-6′′′) to 170.7, 4.56 (1H, m, H-2′′′′′′) to δ_C_ 169.8, 4.08/4.05 (2H, m, H_2_-6′′′′′′) to 170.7. The key HMBC correlations of compound **6** were showed in Figure 2. All the protons and carbons were well assigned by NMR analysis. Therefore, compound **6** was determined as 1-(β-d-glucopyranosyloxybenzyl-6′′′-acetyl)-2-(β-d-glucopyranosyl-2′′′′′′,6′′′′′′-diacetyl)-4-(β-d-gluco pyranosyloxybenzyl)-2*R*-benzylmalate, and named Arundinoside J.

The molecular formula of compound **5** was determined to be C_49_H_60_O_25_ based on the HR-ESI-MS ion [M + NH_4_]^+^ at *m*/*z* 1066.3766. ^1^H and ^13^C NMR data of compound 5 indicated that it was a glucosyloxybenzyl 2*R*-benzylmalate derivative with three acetyl groups as the same as compound 6, but one group substituted position was different. The structure of compound **5** was further confirmed by HSQC and HMBC experiments. The substituent positions of three acetyl groups were determined at C-2′′′′′′, C-4′′′′′′ and C-6′′′′′′ according to HMBC correlations from δ_H_ 4.68 (1H, m, H-2′′′′′′) to δ_C_ 169.7, 4.64 (1H, m, H-4′′′′′′) to 169.3, 4.05/3.76 (2H, m, H_2_-6′′′′′′) to 170.0. Therefore, compound **5** was identified as 1-(β-d-glucopyranosyloxybenzyl)-2-(β-d-glucopyranosyl-2′′′′′′,4′′′′′′,6′′′′′′-triacetyl)-4-(β-d-glucopyranosyloxybenzyl)-2*R*-benzylmalate, and named Arundinoside I.

The molecular formula of compound **8** was determined to be C_51_H_62_O_26_ based on the HR-ESI-MS ion [M + NH_4_]^+^ at *m*/*z* 1108.3869. ^1^H and ^13^C NMR data indicated the structure of compound **8** was a glucosyloxybenzyl 2*R*-benzylmalate derivative with four acetyl groups. Further analysis of HMBC correlations from δ_H_ 4.08/4.27 (2H, m, H_2_-6′′′) to 170.7, 4.69 (1H, m, H-2′′′′′′) to 169.6, 4.93 (1H, m, H-3′′′′′′) to 170.3, 4.05/4.08 (2H, m, H-6′′′′′′) to 170.1 suggested that four acetyl groups of compound **8** substituted at C-6′′′, C-2′′′′′′, C-3′′′′′′, C-6′′′′′′, respectively. Therefore, compound **8** was identified as 1-(β-d-glucopyranosyloxybenzyl-6′′′-acetyl)-2-(β-d-glucopyranosyl-2′′′′′′,3′′′′′′,6′′′′′′-triacetyl)-4-(β-d-glucopyranosyloxybenzyl)-2*R*-benzylmalate, and named Arundinoside K.

The molecular formula of compound **2** was determined to be C_43_H_54_O_22_ based on the HR-ESI-MS ion [M + NH_4_]^+^ at *m*/*z* 940.3453. ^1^H and ^13^C NMR data showed compound **2** was a glucosyloxybenzyl 2-benzylmalate derivative without acetyl group, and its structure was further confirmed by HSQC and HMBC experiments. Therefore, compound **2** was identified as 1-(β-d-glucopyranosyloxybenzyl)-2-(β-d-glucopyranosyl)-4-(β-d-glucopyranosyloxy-benzyl)-2*R*-benzylmalate, and named Arundinoside H.

### 2.2. MS Fragmentation Pattern

HPLC-ESI-MS^n^ experiments were carried out to analysis structural characterization and discuss the fragmentation behaviors of glucosyloxybenzyl 2*R*-benzylmalates **1**–**8** from *A. graminifolia*. The target glucosyloxybenzyl 2*R*-benzylmalates recorded at retention times were designed as A1–A6, B1–B6, C1–C3, D1–D6. The positive ion mode was performed on each of these components, and ESI-MS^n^ data were summarized in Table 3 and Table 4. 

#### 2.2.1. MS Fragmentation Pathway of Glucosyloxybenzyl 2*R*-Benzylmalate Derivatives Isolated

**Figure 3:**
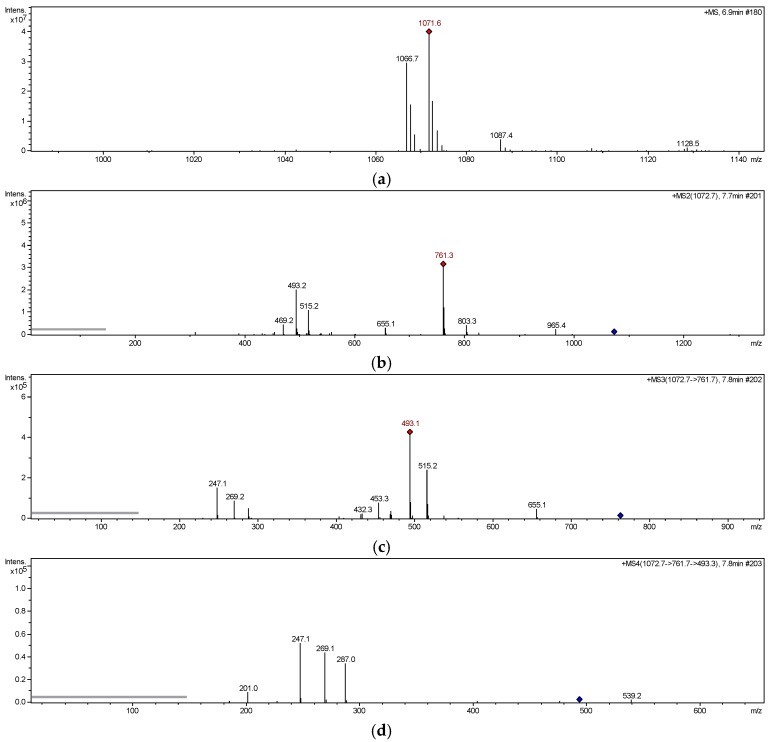
MS^n^ spectra of compound **6**. (**a**) Full-scan MS^1^ spectrum, (**b**) ESI-MS^2^ spectrum, (**c**) ESI-MS^3^ spectrum, (**d**) ESI-MS^4^ spectrum.

**Figure 4:**
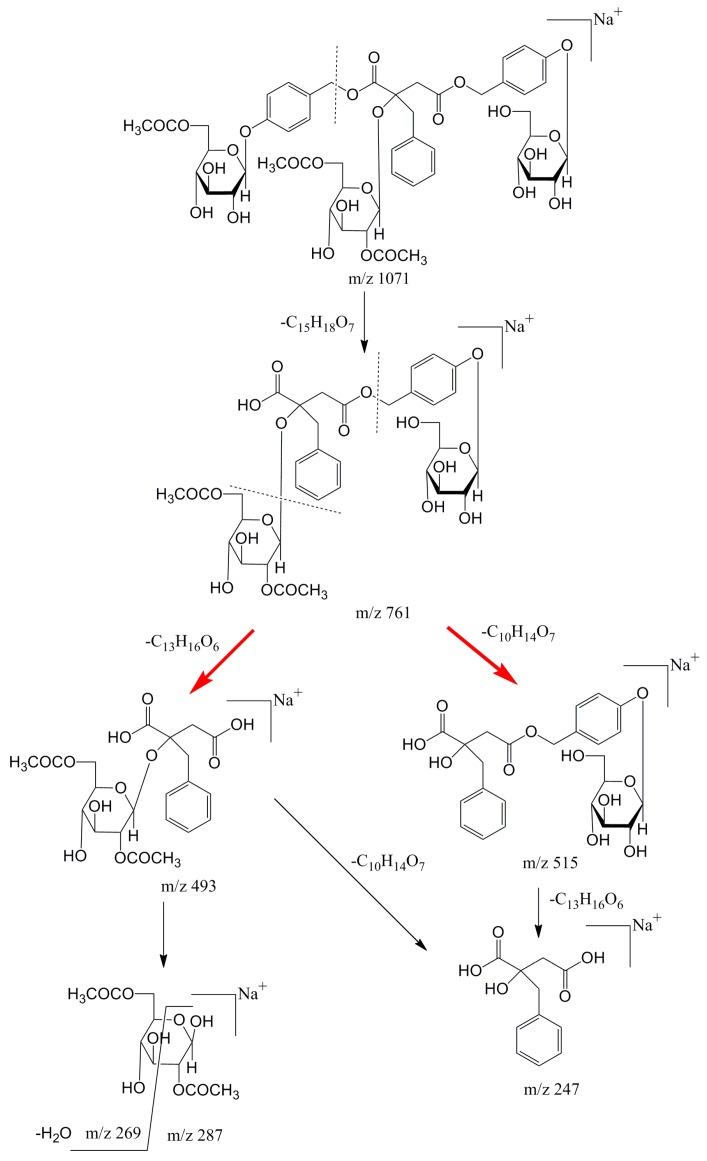
Proposed MS fragment pathway of compound **6**.

In ESI-MS^1^ spectrum of compound **6** (Figure 3a), significant molecular ion peaks at *m*/*z* 1066 [M + NH_4_]^+^, 1071 [M + Na]^+^,1087 [M + K]^+^ were observed, among which the [M + Na]^+^ and product ions were sufficient abundance for further analysis. In ESI-MS^2^ spectrum of compound **6** (Figure 3b), the ion at *m*/*z* 761 was produced by loss of 6′′′-acetyl-5″-*O*-glucosyl-benzyl (CH_2_-Ph-O-Glc-Ac, 310 Da) from parent ion [M + Na]^+^. In ESI-MS^3^ spectrum of compound **6** (Figure 3c), the ions at *m*/*z* 515 and 493 were generated by losing 2′′′′′′,6′′′′′′-diacetyl-glucosyl (Glc-2Ac, 246 Da) and 5′′′′-*O*-glucosyl benzyl (CH_2_-Ph-O-Glc, 268 Da) from *m*/*z* 761, respectively. In ESI-MS^4^ spectrum of compound **6** (Figure 3d), the fragment at *m*/*z* 247, 287, 269 were obviously observed. The ion at *m*/*z* 247 could be produced by ions at *m*/*z* 493 or 515, which suggested that the basic structure of compound 6 was 2-benzyl-malic acid. The ions at *m*/*z* 287 and 269 were obtained by loss of 2-benzyl-malic acid (C_11_H_10_O_4_, 206 Da) and water molecule (H_2_O, 18 Da) successively from *m*/*z* 493. Figure 4 showed the proposed fragmentation pathway of compound **6 [20]**. The same rules were found in the MS^n^ analysis of other isolates listed in Table 3. 

#### 2.2.2. Structural Prediction of Glucosyloxybenzyl 2*R*-Benzylmalates Unreported

We also examined unknown glucosyloxybenzyl 2*R*-benzylmalate derivatives in fractions Ⅰ~Ⅵ of *n*-BuOH extract with CH_3_CN-H_2_O (32:68, *v*/*v*) for mass spectrometry analysis. In the MS^n^ spectra, similar fragmentation pathways as described above were observed, and the possible structures of chemical components A1-A6, B1-B6, C1-C3, D1-D6 were inferred (Table 4). Herein, the analytic procedures were explained by peak A8 and A5. 

**Figure 5:**
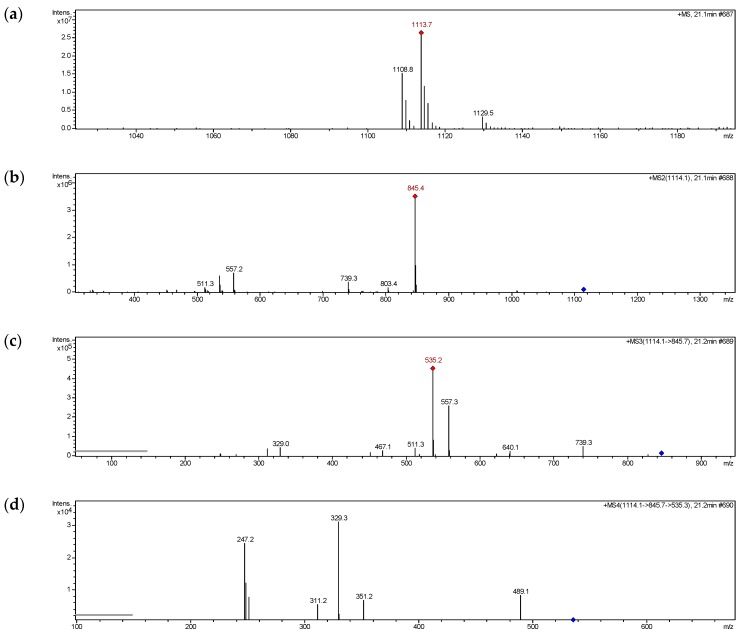
MS^n^ spectra of A8. (**a**) Full-scan MS^1^ spectrum, (**b**) ESI-MS^2^ spectrum, (**c**) ESI-MS^3^ spectrum, (**d**) ESI-MS^4^ spectrum.

**Figure 6:**
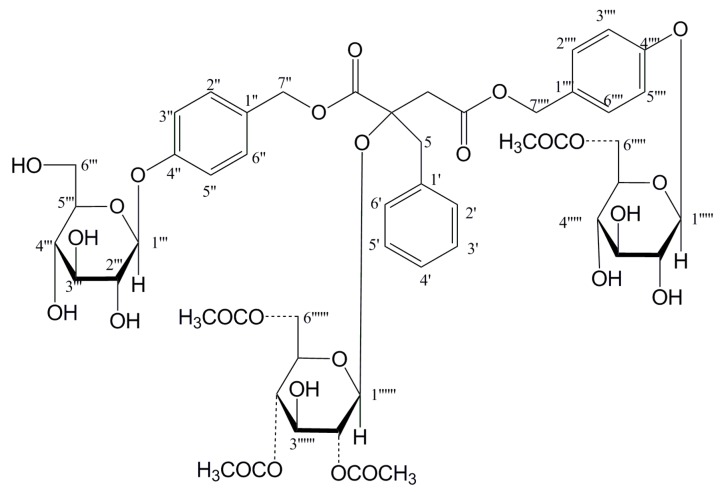
Proposed structure of A8.

The mass spectra of A8 contained significant ions at *m*/*z* 1108 [M + NH_4_]^+^, 1113 [M + Na]^+^, 1129 [M + K]^+^ (Figure 5a). Neutral loss of 268 Da (CH_2_-Ph-O-Glc) and 310 Da (CH_2_-Ph-O-Glc-Ac) were obtained from precursor ion at *m*/*z* 1113 to produce fragment ions at *m*/*z* 845 and *m*/*z* 535 in succession (Figure 5b,c). Then, the ion at *m*/*z* 329 obtained by loss of C_11_H_10_O_4_ (206 Da) from *m*/*z* 535, combining the ion at *m/z* 247 (Figure 5d), indicated the presence of 2-benzyl-malic acid moiety in A8. Based on its fragmentation behaviors and previous studies, A8 was inferred to be the structure shown in Figure 6. Moreover, the mass spectra of A5 showed the same molecular formula and similar fragmentation ions with A8, but the retention time on HPLC with the same conditions were different, which indicated A5 was an isomer of A8. The succession of neutral loss of 310 Da and 268 Da obtained from the ion at *m*/*z* 845 produced by precursor ion at *m*/*z* 1113 suggested one of Ac groups in A5 was located at G2, and not at G1. The same experimental procedures were applied to analyze other molecules list in Table 4, and the main fragments observed in MS^n^ spectra of the [M + Na]^+^ precursor ions were summarized. 

### 2.3. Anti-hepatic Fibrosis Activity

**Figure 7:**
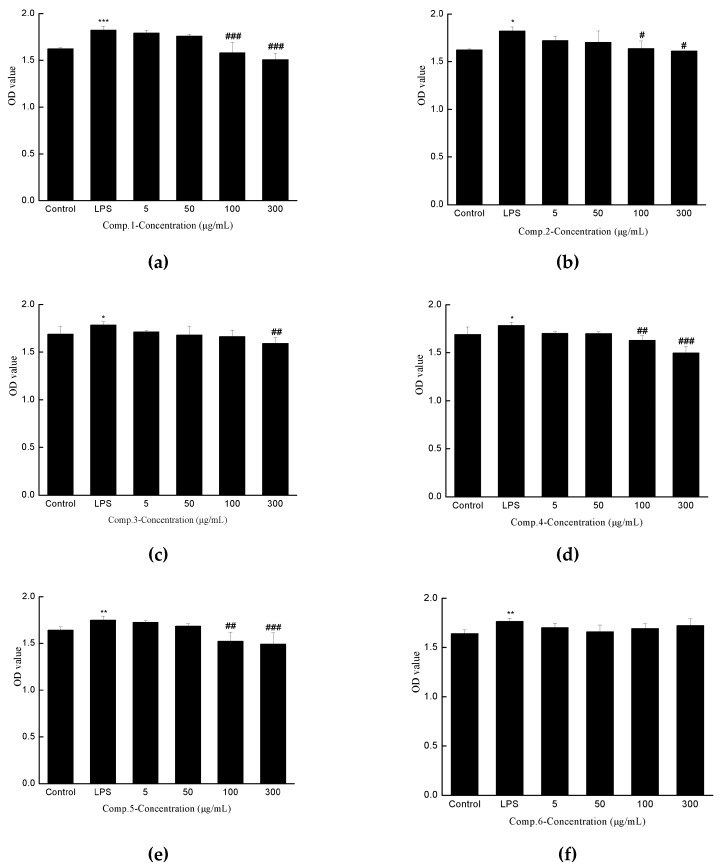

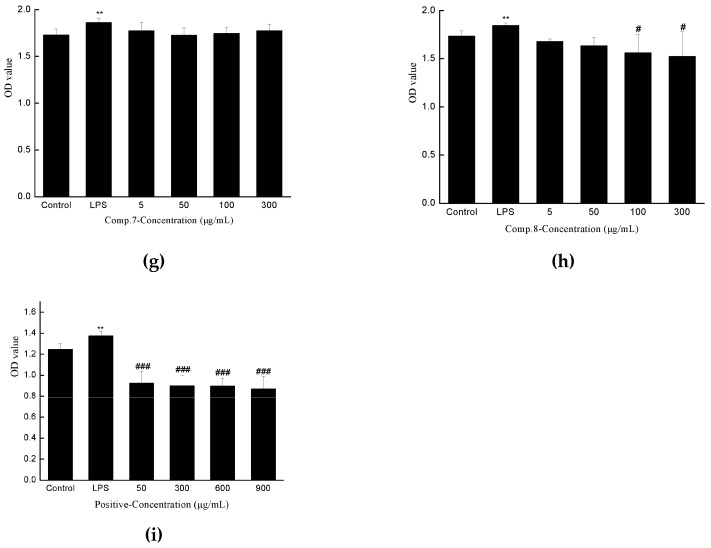
Inhibitory activity of compound **1** (**a**)–**8** (**h**) and positive control (**i**) on the proliferation of LPS-activated HSC-T6.

Emerging studies indicated that HSC in resting state could be induced to activated state by LPS [21], while the inhibition of proliferation of activated HSC, has been considered as an effective target for liver fibrosis [22]. In addition, Considering the bioactive results obtained for the “BaoGan capsule” in our previous work, the anti-hepatic fibrosis activities of the isolates 1–8 were tested on the proliferation of LPS-activated HSC-T6 cells in vitro by MTS method. Legalon (silymarin capsules) was taken as a positive control. As shown in Figure 7, compound **1**, **2**, **4**, **5**, **8** were exhibited moderate anti-proliferative activity with significantly different values while the concentration was 100 μg/mL, while positive control showed a significant difference at 50 μg/mL.

## 3. Materials and Methods 

### 3.1. General Experimental Procedures

HPLC analyses were performed on Shimadzu LC-20AD (Shimadzu, Kyoto, Japan) equipped with a ZORBAX Eclipse XDB-C_18_ column (250 × 4.6 mm, 5 μm, Agilent, Santa Clara, CA, USA) and a SPD-20A detector. Preparative HPLC separations were conducted on a Shimadzu LC-6AD system with a preparative reversed-phase C_18_ column (250 × 20 mm, 5 μm, YMC-Pack ODS-A, Tokyo, Japan) and a SPD-6A detector. Mobile phase were purified water and methanol with chromatographic grade, which were bought from Merck. Organic reagents were analytical grade (Beijing Chemical Works, Beijing, China). One dimensional nuclear magnetic resonance (1D NMR: ^1^H, ^13^C, DEPT) and two dimensional nuclear magnetic resonance (2D NMR: HSQC, HMBC, ^1^H-^1^HCOSY) were measured on Bruker 700MHz AVANCE III spectrometer and Bruker AVANCE DRX-500 spectrometer (Karlsruhe, Germany) in DMSO-d_6_ (Sigma-Aldrich, St. Louis, MO, USA). Chemical shifts are shown in δ (ppm) with tetramethylsilane (TMS) as an internal standard. High resolution-electrospray ionization-mass spectrometry (HR-ESI-MS) and High-performance liquid chromatography-electrospray ionization- multiple stage mass spectrometry (HPLC-ESI-MS^n^) data were obtained from a 1100 Agilent Series coupled to an Agilent 6520 Accurate Mass Q-TOF and LC-MSD trap Mass spectrometer (Agilent Technologies, Santa Clara, CA, USA), respectively.

### 3.2. Plant Material

The whole plant of *A. graminifolia* was bought from Dai hospital of Xishuanbanna Autonomous Prefecture, Yunnan Province, China. A voucher specimen (batch number: 20111128) was collected in the laboratory.

### 3.3. Extraction and Isolation

Air dried powder of *A. graminifolia* (8.0 kg) was decocted with 80% ethanol (3 times, 2 h/time) at room temperature and extracting solution was merged and filtered. The filtrate was evaporated under reduced pressure to acquire crude extraction, which was further extracted with petroleum ether, chloroform, ethyl acetate and n-butanol to obtain the corresponding fractions. The *n*-BuOH extract was fractioned on a macroporous resin adsorption column eluting with ethanol/water (10:90, 50:50, 100:0, *v*/*v*) to yield 3 fractions (A–C). Fraction B (22.2 g) was subjected to Rp-18 silica gel column eluted with acetonitrile/water (10:90→100:0, *v*/*v*) to obtain five fractions (B1–B5). Fraction B3 **(**5.5 g**)** was then separated by silica gel column eluted with CHCl_3_/CH_3_OH (20:1, 2:1, 0:1) to give six fractions (Ⅰ–Ⅵ). Fraction Ⅴ (0.48 g) was submitted to preparative HPLC on a Rp-18 column (250 mm × 20 mm, wavelength 279 nm, flow rate 4 mL/min) with CH_3_CN-H_2_O (35:65, *v*/*v*) to give compound **1** (26.83 mg, RT = 21.5 min) and peaks D1~D6 eluted by CH_3_CN-H_2_O with 5 mM ammonium acetate (32:68, *v*/*v*). Fraction Ⅱ (2.64 g) was eluted with CH_3_CN-H_2_O (29:71,*v*/*v*) to afford compound **2** (1.71 mg, RT = 5 min), compound **3** (1.62 mg, RT = 8.2 min) and compound **4** (3.84 mg, RT = 15.2 min), with CH_3_CN-H_2_O (26:74, *v*/*v*) to afford compound **5** (2.34 mg, RT = 25 min), compound **6** (3.18 mg, RT = 30 min) and compound **7** (1.85 mg, RT = 41.9 min), with CH_3_CN-H_2_O (30:70, *v*/*v*) to afford compound **8** (2.01 mg, RT = 42 min). Furthermore, a search of the rest of Fraction Ⅱ was then conducted at 0.2 mL/min for HPLC-ESI-MS^n^ to obtain Peaks A1~A9 eluted by CH_3_CN-H_2_O with 5 mM ammonium acetate (30:70, *v*/*v*), peaks B1~B6 eluted by CH_3_CN-H_2_O with 5 mM ammonium acetate (26:74, *v*/*v*); peaks C1~C3 eluted by CH_3_CN-H_2_O with 5 mM ammonium acetate (27:73, *v*/*v*), and RT values of the peaks were shown in Table 4.

### 3.4. Cell Proliferation Inhibition Assay

#### 3.4.1. Chemical and Reagents

LPS, RPMI-1640 medium, penicillin-streptomycin, and trypsin were bought from Solarbio, Beijing, China and fetal bovine serum were purchased from Sijiqing, Hangzhou, China. Dimethyl sulfoxide (DMSO), PMS and MTS [3-(4,5-dimethylthiazol-2-yl)-5-(3-carboxymethoxyphenyl)-2-(4- sulfophenyl)-2*H*-tetrazolium, inner salt] were purchased from Sigma-Aldrich (Steinheim, Germany).

#### 3.4.2. In vitro Evaluation of Anti-Liver Fibrotic Activity

HSC-T6 cells were maintained in RPMI-1640 medium supplemented with 10% fetal bovine serum and 1% penicillin-streptomycin in an incubator with constant temperature at 37 °C and a humidified atmosphere of 5% CO_2_. The cells were trypsinized and passaged to new plates every two or three days. HSC-T6 cells were seeded in 96-well plates (5 × 10^3^/100 μL) for 24 h to ensure fully adhesion and good condition. The cell medium in the wells was changed into fresh RPMI-1640 medium supplemented with 5% fetal bovine serum for further incubation. Cells incubated with LPS (1 μg/mL) in the different concentration of compounds **1**~**8** (0, 5, 50, 100, 300 μg/mL) were cultivated for another 24 h. Each group was provided with 6 independent duplicates. Cell viability was determined using MTS/PMS assay. Absorbance values were read at 490 nm on an ELISA reader. 0.1% DMSO was considered as blank control and legalon (silymarin capsules) as positive control. Cell viability was expressed as a percentage of control cells at 100% viability. Statistical analysis was performed using origin Pro 8.0 (OriginLab Corpration, One Roundhouse Plaza, Northampton, MA, USA).

## 4. Conclusions

Glucosyloxybenzyl 2*R*-benzylmalates are a class of naturally occurring substances distributed in Orchidaceae. They were noticed for their novel type of structure and significant activities, while the research of glucosyloxybenzyl 2*R*-benzylmalates was limited by their higher polarity and less content. In present work, basis on the information acquired from HPLC-ESI-MS^n^ experiment combined with NMR analysis, it was possible not only to identify 8 compounds isolated from *A. graminifolia*, but also to predict the structures of 24 previously unreported glucosyloxybenzyl 2*R*-benzylmalates in the extract. The ESI-MS^n^ experiments provide a useful guide for gaining the large structural information of novel compounds, which are important to drug design, although the analytical method cannot confirm the accurate substitution position of Ac groups of these glucosyloxybenzyl 2*R*-benzylmalates. 

In addition, a cell model associated with hepatic fibrosis was established by using LPS to stimulate HSC-T6. The isolates were carried to hepatic fibrosis experiment while compounds **1**, **2**, **4**, **5**, **8** showed moderate anti-hepatic fibrosis effects. Nevertheless, the studies on the quantitative structure-antihepatic fibrosis relationship of predicted glucosyloxybenzyl 2*R*-benzylmalates will be further investigated. 

## 5. Patents

Two patents resulting from the work about new structures, anti-liver fibrotic activity and MS fragment pathway have been submitted to the Chinese Patent Office.

## Figures and Tables

**Table 1:** ^1^H nuclear magnetic resonance (NMR) data of four new compounds (700 MHz, DMSO-d6).

Position	2	5	6	8
3	2.96 (d, 17.7); 2.82 (d, 17.7)	2.96 (d, 15); 2.90 (d, 15)	2.96 (d, 17.8); 2.90 (d, 17.8)	2.96 (d, 17.8); 2.92 (d, 17.8)
5	3.17 (m); 3.06 (m)	3.10 (d, 14); 3.02 (d, 14)	3.10(d, 14); 3.02 (d, 14)	3.10 (d, 14); 3.02 (d, 14)
2′, 6′	7.19 (m)	7.18 (m)	7.18 (m)	7.18 (m)
3′, 5′	7.16 (m)	7.02 (m)	7.01 (d, 8.7)	7.01 (d, 8.7)
4′	7.19 (m)	7.18 (m)	7.17 (m)	7.17 (m)
1″	4.99 (d, 7.9); 4.90 (d, 7.9)	5.00 (d, 12); 4.92 (d, 12)	5.00 (d, 12); 4.93 (d,12)	5.00(d,12); 4.93 (d,12)
3″, 7″	7.27 (d, 8.4)	7.27 (d, 8.3)	7.27 (d, 8.7)	7.27 (d, 8.7)
4″, 6″	7.01 (d, 8.5)	7.02 (m)	7.01 (d, 8.7)	7.01 (d, 8.7)
Glc-1′′′	4.87 (d, 7.5)	4.91 (d, 7.9)	4.92 (d, 7.5)	4.92 (d, 7.7)
Glc-2′′′	3.27 (m)	3.26 (m)	3.27 (m)	3.27 (m)
Glc-3′′′	3.24 (m)	3.23 (m)	3.22 (m)	3.22 (m)
Glc-4′′′	3.16 (m)	3.16 (m)	3.18 (m)	3.18 (m)
Glc-5′′′	3.27 (m)	3.26 (m)	3.27 (m)	3.27 (m)
Glc-6′′′	3.68 (m); 3.47 (m)	3.68 (m); 3.46 (m)	4.27 (m); 4.08 (m)	4.27 (m); 4.08 (m)
Glc-6′′′-COCH_3_	-	-	1.99 (s)	1.99 (s)
1′′′′	5.01 (d, 12); 4.90 (d, 12)	5.04 (d, 12); 4.91 (d, 12)	5.00 (d, 12); 4.99 (d, 12)	5.00 (d,12); 4.99 (d,12)
3′′′′, 7′′′′	7.25 (d, 8.4)	7.23 (d, 8.3)	7.23 (d, 8.7)	7.23 (d, 8.7)
4′′′′, 6′′′′	7.02 (d, 8.5)	7.02 (m)	7.02 (d, 8.7)	7.02 (d, 8.7)
Glc-1′′′′′	4.87 (d, 7.5)	4.86 (d, 7.7)	4.92 (d, 7.5)	4.87 (d, 7.5)
Glc-2′′′′′	3.27 (m)	3.16 (m)	3.30 (m)	3.30 (m)
Glc-3′′′′′	3.24 (m)	3.23 (m)	3.22 (m)	3.22 (m)
Glc-4′′′′′	3.16 (m)	3.16 (m)	3.18 (m)	3.18 (m)
Glc-5′′′′′	3.27 (m)	3.26 (m)	3.27 (m)	3.27 (m)
Glc-6′′′′′	3.69 (m); 3.47 (m)	3.68 (m); 3.46 (m)	3.68 (m); 3.46 (m)	3.68 (m); 3.46 (m)
Glc-1′′′′′′	4.66 (d, 7.8)	4.94 (d, 8.0)	4.86 (d, 7.6)	5.05 (d, 8.0)
Glc-2′′′′′′	3.03 (m)	4.68 (m)	4.56 (m)	4.69 (m)
Glc-3′′′′′′	3.39 (m)	3.46 (m)	3.68 (m)	4.93 (m)
Glc-4′′′′′′	4.36 (m)	4.64 (m)	3.46 (m)	3.46 (m)
Glc-5′′′′′′	3.52 (m)	3.45 (m)	3.61 (m)	3.61 (m)
Glc-6′′′′′′	3.68 (m); 3.38 (m)	4.05 (m); 3.76 (m)	4.08 (m); 4.05 (m)	4.08 (m); 4.05 (m)
Glc-2′′′′′′-COCH_3_	-	1.70 (s)	1.72 (s)	1.65 (s)
Glc-3′′′′′′-COCH_3_	-	-	-	1.92 (s)
Glc-4′′′′′′-COCH_3_	-	2.01 (s)	-	
Glc-6′′′′′′-COCH_3_	-	1.92 (s)	1.92 (s)	1.99 (s)

**Table 2:** ^13^C NMR data of four new compounds (175MHz, DMSO-d6).

Position	2	5	6	8
1	170.9	170.1	170.5	170.1
2	79.8	80.7	81.0	81.3
3	40.4	40.9	41.0	41.0
4	169.8	169.5	170.1	170.1
5	42.0	43.6	43.8	43.7
1′	135.5	135.0	135.5	135.4
2′, 6′	127.9	127.9	128.3	128.3
3′, 5′	130.6	130.5	130.9	130.9
4′	126.7	126.7	127.1	127.1
1″	66.3	66.2	66.6	66.6
2″	128.9	128.8	129.3	129.2
3″, 7″	129.9	129.9	130.3	130.3
4″, 6″	116.2	116.2	116.7	116.7
5″	157.4	157.4	157.9	157.9
Glc-1′′′	100.3	100.3	100.5	100.5
Glc-2′′′	76.8	76.6	77.1	77.1
Glc-3′′′	73.2	73.2	73.5	73.6
Glc-4′′′	69.6	69.7	70.1	70.2
Glc-5′′′	76.6	76.6	76.8	76.8
Glc-6′′′	60.7	60.7	63.9	63.8
Glc-6′′′-COCH_3_	-	-	170.7; 21.2	170.7; 21.1
1′′′′	65.6	65.7	66.2	66.2
2′′′′	128.8	128.6	129.3	129.3
3′′′′, 7′′′′	129.9	129.9	130.4	130.4
4′′′′, 6′′′′	116.1	116.2	116.5	116.5
5′′′′	157.4	157.4	157.6	157.6
Glc-1′′′′′	100.3	100.4	100.8	100.8
Glc-2′′′′′	76.8	77.0	77.5	77.5
Glc-3′′′′′	73.2	73.2	73.6	73.4
Glc-4′′′′′	69.6	70.3	70.1	70.1
Glc-5′′′′′	76.6	76.6	76.8	76.8
Glc-6′′′′′	60.7	60.7	61.1	61.1
Glc-1′′′′′′	98.3	96.7	97.0	96.7
Glc-2′′′′′′	77.0	70.9	73.8	71.2
Glc-3′′′′′′	73.7	70.9	74.0	75.1
Glc-4′′′′′′	69.7	72.8	73.7	67.9
Glc-5′′′′′′	76.7	70.6	74.1	74.1
Glc-6′′′′′′	61.2	61.8	63.3	62.9
Glc-2′′′′′′-COCH_3_	-	169.7; 20.5	169.8; 21.0	169.6; 20.7
Glc-3′′′′′′-COCH_3_	-	-	-	170.3; 21.0
Glc-4′′′′′′-COCH_3_	-	169.3; 20.8	-	-
Glc-6′′′′′′-COCH_3_	-	170; 20.7	170.7; 21.1	170.1;21.2

**Table 3:** HR-ESI-MS and key ESI-MS^n^ data of the isolates **1**–**8**.

Compounds	Molecular Formula	HR-ESI-MS [M + NH_4_]^+^	ESI-MS^1^: [M + Na]^+^	ESI-MS^n^
1	C_51_H_62_O_26_	1108.3861	1113	845, 577, 515, 497, 371, 353, 311, 251, 247
2	C_43_H_54_O_22_	940.3453	945	677, 515, 409, 247
3	C_45_H_56_O_23_	982.3554	987	719, 515, 451, 247
4	C_47_H_58_O_24_	1024.3665	1029	761, 515, 493, 287, 269, 247, 227
5	C_49_H_60_O_25_	1066.3766	1071	803, 535, 329, 311, 269, 247
6	C_49_H_60_O_25_	1066.3764	1071	761, 515,493, 287, 269, 247
7	C_49_H_60_O_25_	1066.3766	1071	803, 535, 515, 329, 311, 247, 209
8	C_51_H_62_O_26_	1108.3869	1113	803, 535, 515, 329, 311, 247, 209

**Table 4:**
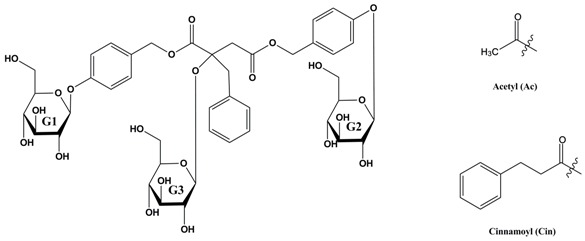
Key ESI-MS^n^ Fragment Ions and structural information of the components predicted.

Peaks	RT	MS^1^ [M + Na]^+^	MS^n^	G1	G2	G3
A1	3.9 min	1006	1029, 761, 515, 493, 287, 269, 247			2Ac
A2	6.4 min	1048	1071, 803, 535, 515, 329, 311			3Ac
A3	7.3 min	1048	1072, 761, 515, 493, 287, 269, 247	Ac		2Ac
A4	12.7 min	1090	1113, 845, 535, 329, 311		Ac	3Ac
A5	13.9 min	1090	1113, 803, 535, 329, 311, 247	Ac		3Ac
A6	14.0 min	1052	1071, 677, 515, 247	Cin		
A7	22.2 min	1094	1117, 849, 645, 247		Cin	Ac
A8	21.1 min	1090	1113, 845, 535, 329, 247		Ac	3Ac
A9	28.0 min	1094	1117, 719, 451, 247	Cin		Ac
B1	3.7 min	964	987, 719, 515, 247			Ac
B2	4.3 min	964	987, 719, 515, 247			Ac
B3	7.0 min	760	783, 515, 247			OH
B4	9.0 min	1006	1029, 761, 557, 451, 247		Ac	Ac
B5	11.0 min	1006	1029, 719, 515, 451, 247	Ac		Ac
B6	12.6 min	1006	1029, 761, 515, 493, 287, 269, 247			2Ac
C1	6.4 min	1048	1071, 761, 557, 451, 247	Ac	Ac	Ac
C2	8.9 min	1048	1071, 761, 515, 493, 287, 269, 247	Ac		2Ac
C3	11.9 min	1048	1071, 803, 535, 329, 311, 269, 247			3Ac
D1	16.0 min	1252	1275, 845, 577, 371, 353, 311			4Ac
D2	8.5 min	1052	1075, 677, 515, 247	Cin		
D3	7.0 min	1052	1075, 677, 515, 247	Cin		
D4	15.6 min	1094	1117, 719, 515, 451, 247	Cin		Ac
D5	18.6 min	1094	1117, 719, 515, 451, 247	Cin		Ac
D6	19.3 min	1094	1117, 719, 515, 451, 247	Cin		Ac

## Supplementary Materials

The following are available online: ^1^H and ^13^C NMR, DEPT135, HSQC, HMBC, ^1^H-^1^H COSY spectra of new compounds **2**, **5**, **6**, and **8**; HPLC-ESI-MS spectra of the isolates **1**–**8**; HPLC-ESI-MS^n^ spectra of peaks A1–A9, B1–B6, C1–C3, and D1–D6.
