# Supplementary material for: HPLC-ESI-MSn Identification and NMR Characterization of Glucosyloxybenzyl 2R-Benzylmalate Deriva-Tives from Arundina Graminifolia and Their Anti-Liver Fibrotic Effects In Vitro
Source: Molecules. 2019 Jan 31;24(3):525. doi: 10.3390/molecules24030525 (PMC6385127; doi:10.3390/molecules24030525)
Supplement: Supplementary file 1 [file molecules-24-00525-s001.pdf]

# Supplementary Materials: HPLC-ESI-MS$^n$ identification and NMR characterization of glucosyloxybenzyl 2*R*-benzylmalate derivatives from *Arundina graminifolia* and their anti-liver fibrotic effects *in vitro*

**Qingqing Liu [1], Feiyi Sun [1], Rongji Dai [1], Yulin Deng [1] and Fang Lv [1,*]**

[1] Beijing Key Labrotary for Separation and Analysis in Biomedicine and Pharmaceuticals, School of Life Science, Beijing Institute of Technology, Beijing 100081, P.R. China
* Correspondence: lvfangbeijing@bit.edu.cn; Tel.: +86-010-68949331

**Supplementary Materials:**

Figure S1. $^1$H-NMR spectrum (700 MHz, DMSO-$d_6$) of Arundinoside H (**2**).
Figure S2. $^{13}$C-NMR spectrum (175 MHz, DMSO-$d_6$) of Arundinoside H (**2**).
Figure S3. DEPT135 spectrum (175 MHz, DMSO-$d_6$) of Arundinoside H (**2**).
Figure S4. HSQC spectrum of Arundinoside H (**2**).
Figure S5. HMBC spectrum of Arundinoside H (**2**).
Figure S6. $^1$H-$^1$H COSY spectrum of Arundinoside H (**2**).

Figure S7. $^1$H-NMR spectrum (700 MHz, DMSO-$d_6$) of Arundinoside I (**5**).
Figure S8. $^{13}$C-NMR spectrum (175 MHz, DMSO-$d_6$) of Arundinoside I (**5**).
Figure S9. DEPT135 spectrum (175 MHz, DMSO-$d_6$) of Arundinoside I (**5**).
Figure S10. HSQC spectrum of Arundinoside I (**5**).
Figure S11. HMBC spectrum of Arundinoside I (**5**).
Figure S12. $^1$H-$^1$H COSY spectrum of Arundinoside I (**5**).

Figure S13. $^1$H-NMR spectrum (700 MHz, DMSO-$d_6$) of Arundinoside J (**6**).
Figure S14. $^{13}$C-NMR spectrum (175 MHz, DMSO-$d_6$) of Arundinoside J (**6**).
Figure S15. DEPT135 spectrum (175 MHz, DMSO-$d_6$) of Arundinoside J (**6**).
Figure S16. HSQC spectrum of Arundinoside J (**6**).
Figure S17. HMBC spectrum of Arundinoside J (**6**).
Figure S18. $^1$H-$^1$H COSY spectrum of Arundinoside J (**6**).

Figure S19. $^1$H-NMR spectrum (700 MHz, DMSO-$d_6$) of Arundinoside K (**8**).
Figure S20. $^{13}$C-NMR spectrum (175 MHz, DMSO-$d_6$) of Arundinoside K (**8**).
Figure S21. DEPT135 spectrum (175 MHz, DMSO-$d_6$) of Arundinoside K (**8**).
Figure S22. HSQC spectrum of Arundinoside K (**8**).
Figure S23. HMBC spectrum of Arundinoside K (**8**).
Figure S24. $^1$H-$^1$H COSY spectrum of Arundinoside K (**8**).

Figure S25. HRESIMS spectrum of Arundinoside D (**1**).

Figure S26. HRESIMS spectrum of Arundinoside H (**2**).

Figure S27. HRESIMS spectrum of Arundinoside G (**3**).

Figure S28. HRESIMS spectrum of Arundinoside F (**4**).

Figure S29. HRESIMS spectrum of Arundinoside I (**5**).

Figure S30. HRESIMS spectrum of Arundinoside J (**6**).

Figure S31. HRESIMS spectrum of Arundinoside E (**7**).

Figure S32. HRESIMS spectrum of Arundinoside K (**8**).

Figure S33. HPLC-ESI-$MS^n$ spectrum of peak **A1**.

Figure S34. HPLC-ESI-$MS^n$ spectrum of peak **A2**.

Figure S35. HPLC-ESI-$MS^n$ spectrum of peak **A3**.

Figure S36. HPLC-ESI-$MS^n$ spectrum of peak **A4**.

Figure S37. HPLC-ESI-$MS^n$ spectrum of peak **A5**.

Figure S38. HPLC-ESI-$MS^n$ spectrum of peak **A6**.

Figure S39. HPLC-ESI-$MS^n$ spectrum of peak **A7**.

Figure S40. HPLC-ESI-$MS^n$ spectrum of peak **A8**.

Figure S41. HPLC-ESI-$MS^n$ spectrum of peak **A9**.

Figure S42. HPLC-ESI-$MS^n$ spectrum of peak **B1**.

Figure S43. HPLC-ESI-$MS^n$ spectrum of peak **B2**.

Figure S44. HPLC-ESI-$MS^n$ spectrum of peak **B3**.

Figure S45. HPLC-ESI-$MS^n$ spectrum of peak **B4**.

Figure S46. HPLC-ESI-$MS^n$ spectrum of peak **B5**.

Figure S47. HPLC-ESI-$MS^n$ spectrum of peak **B6**.

Figure S48. HPLC-ESI-$MS^n$ spectrum of peak **C1**.

Figure S49. HPLC-ESI-$MS^n$ spectrum of peak **C2**.

Figure S50. HPLC-ESI-$MS^n$ spectrum of peak **C3**.

Figure S51. HPLC-ESI-$MS^n$ spectrum of peak **D1**.

Figure S52. HPLC-ESI-$MS^n$ spectrum of peak **D2**.

Figure S53. HPLC-ESI-$MS^n$ spectrum of peak **D3**.

Figure S54. HPLC-ESI-$MS^n$ spectrum of peak **D4**.

Figure S55. HPLC-ESI-$MS^n$ spectrum of peak **D5**.

Figure S56. HPLC-ESI-$MS^n$ spectrum of peak **D6**.

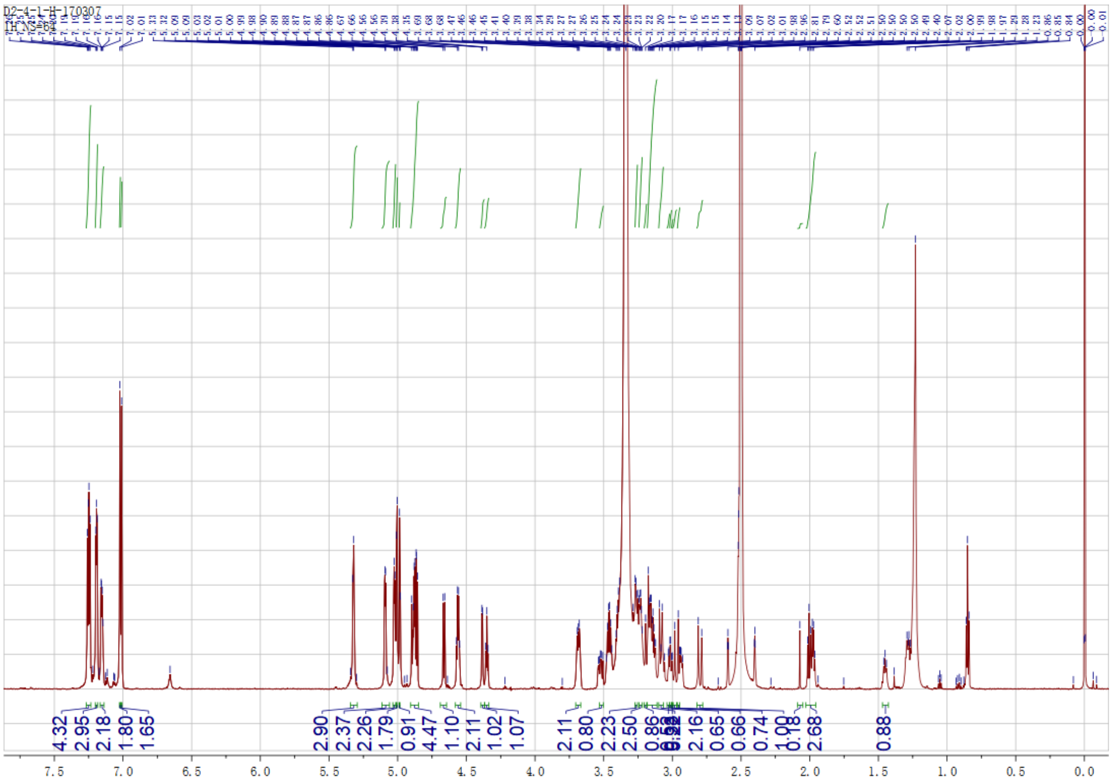


Figure S1. $^{1}$H-NMR spectrum (700 MHz, DMSO-$d_6$) of Arundinoside H (**2**).

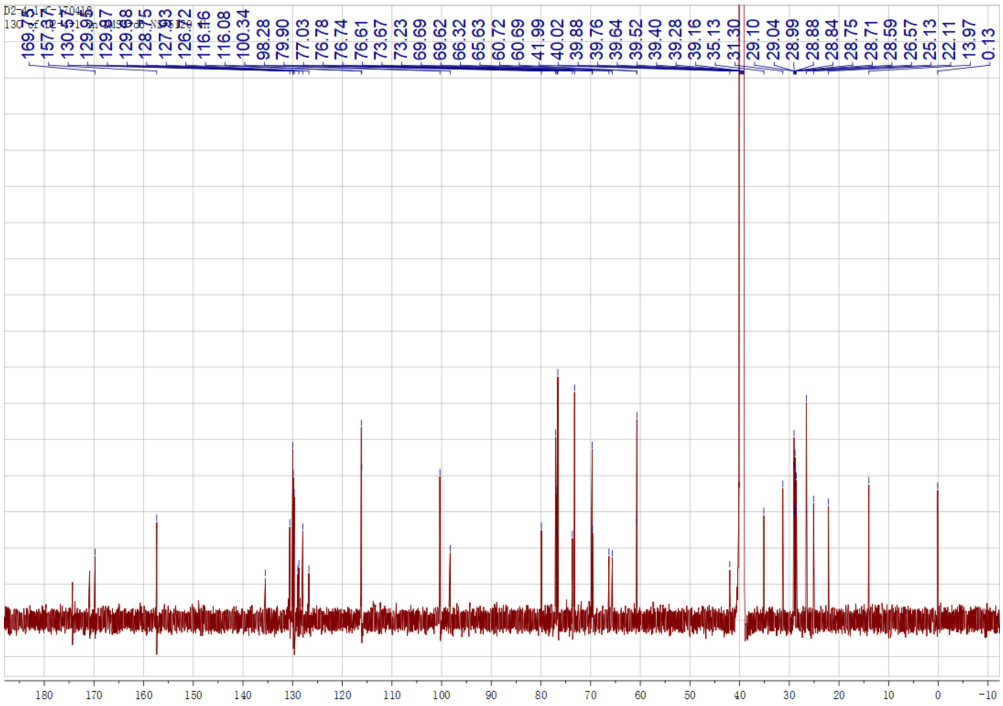


Figure S2. $^{13}$C-NMR spectrum (175 MHz, DMSO-$d_6$) of Arundinoside H (**2**).

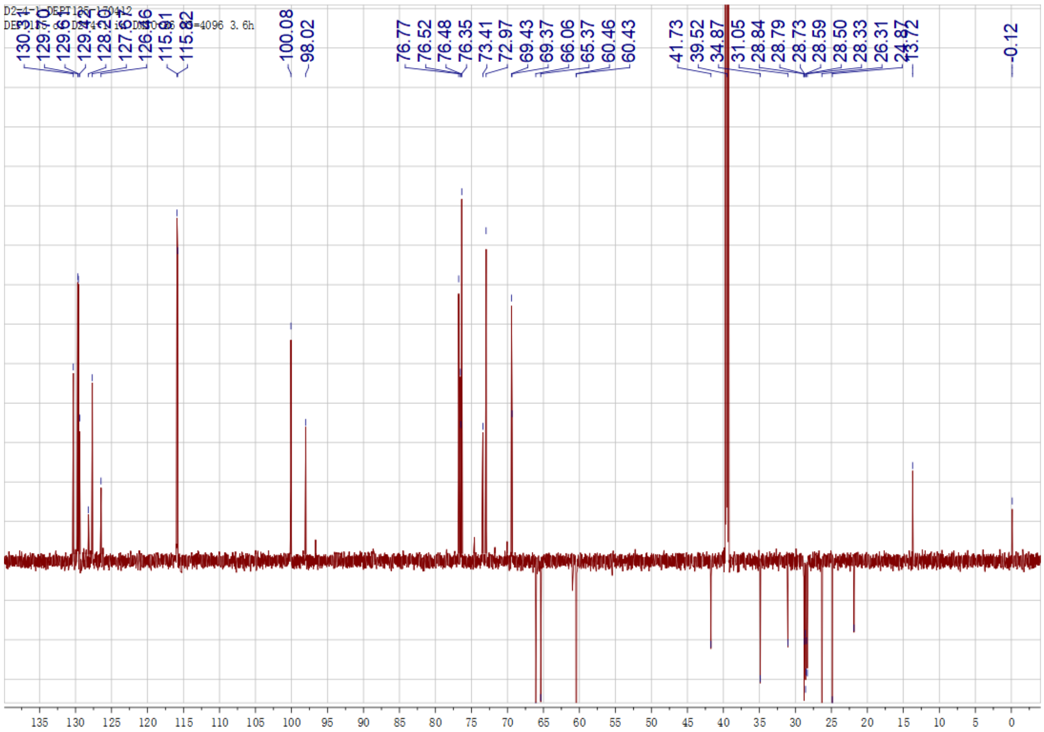


Figure S3. DEPT135 spectrum (175 MHz, DMSO-$d_6$) of Arundinoside H (**2**).

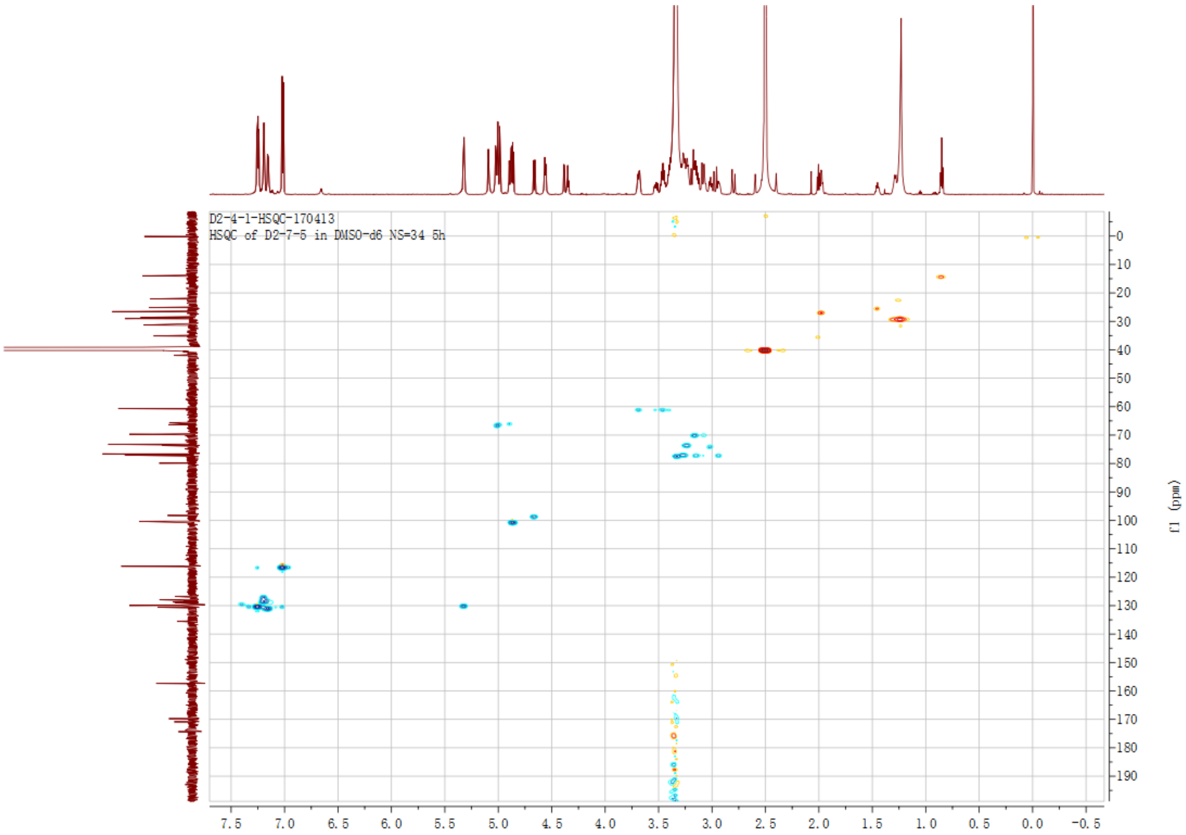


Figure S4. HSQC spectrum of Arundinoside H (**2**).

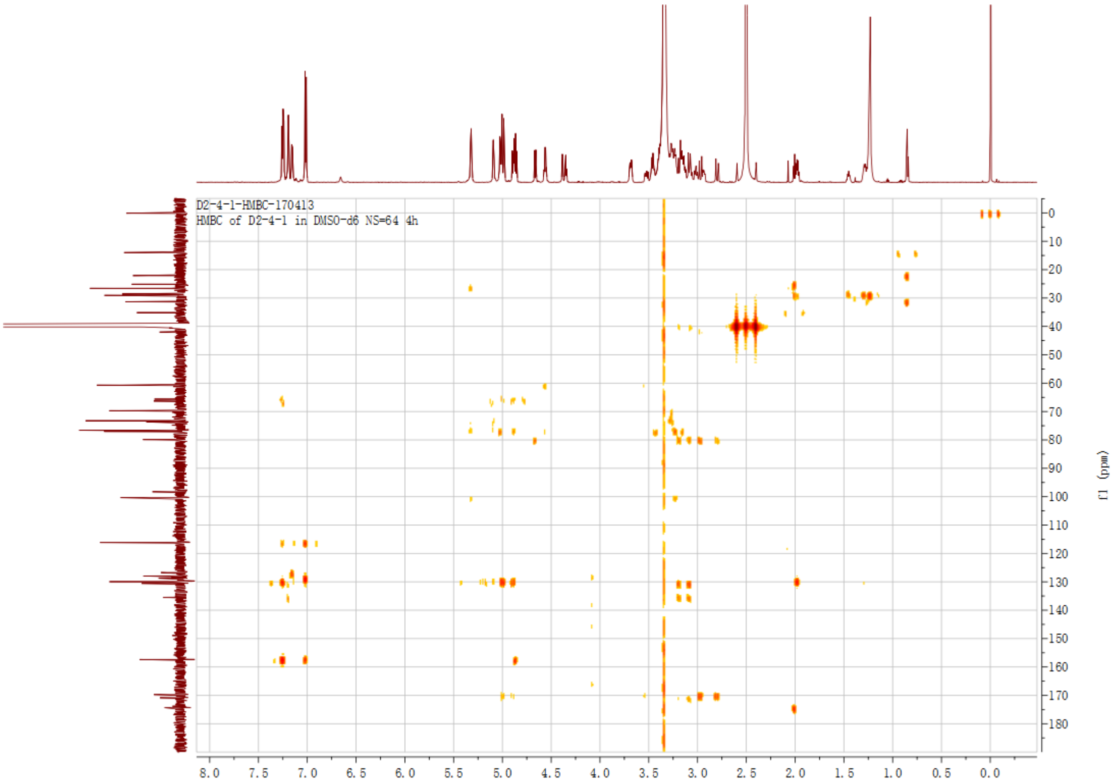


Figure S5. HMBC spectrum of Arundinoside H (**2**).

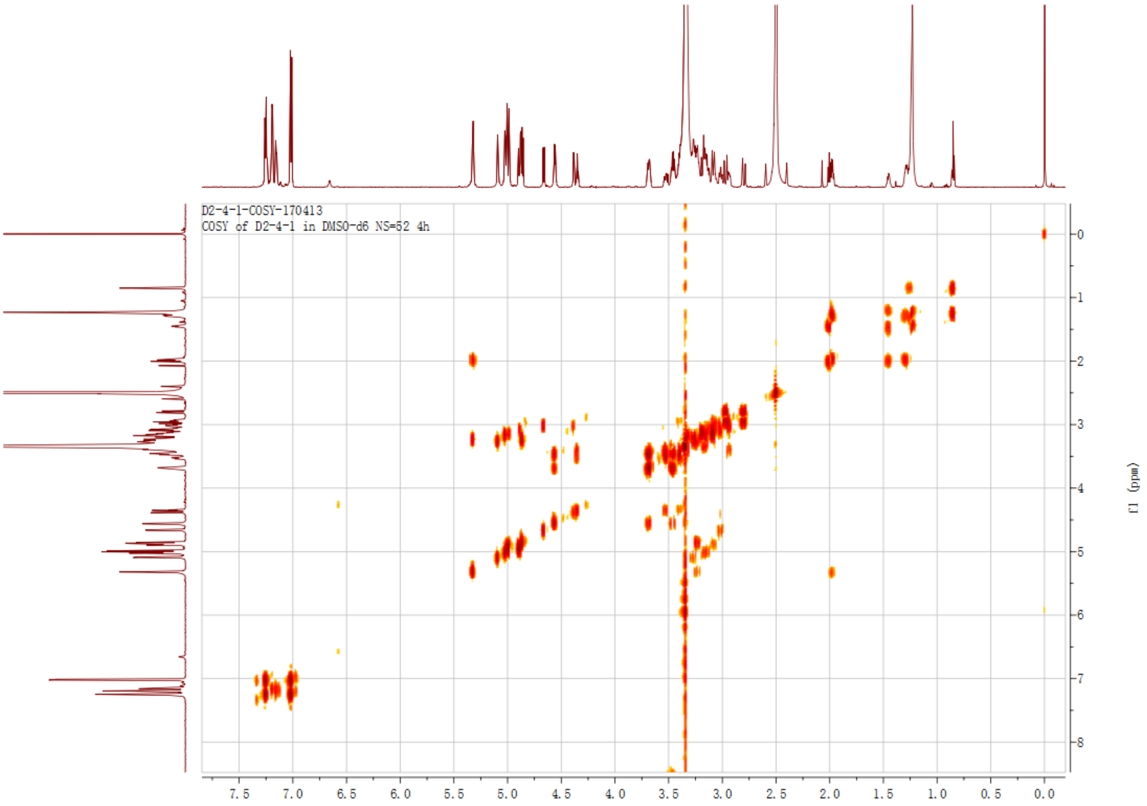


Figure S6. $^{1}H$-$^{1}H$ COSY spectrum of Arundinoside H (**2**).

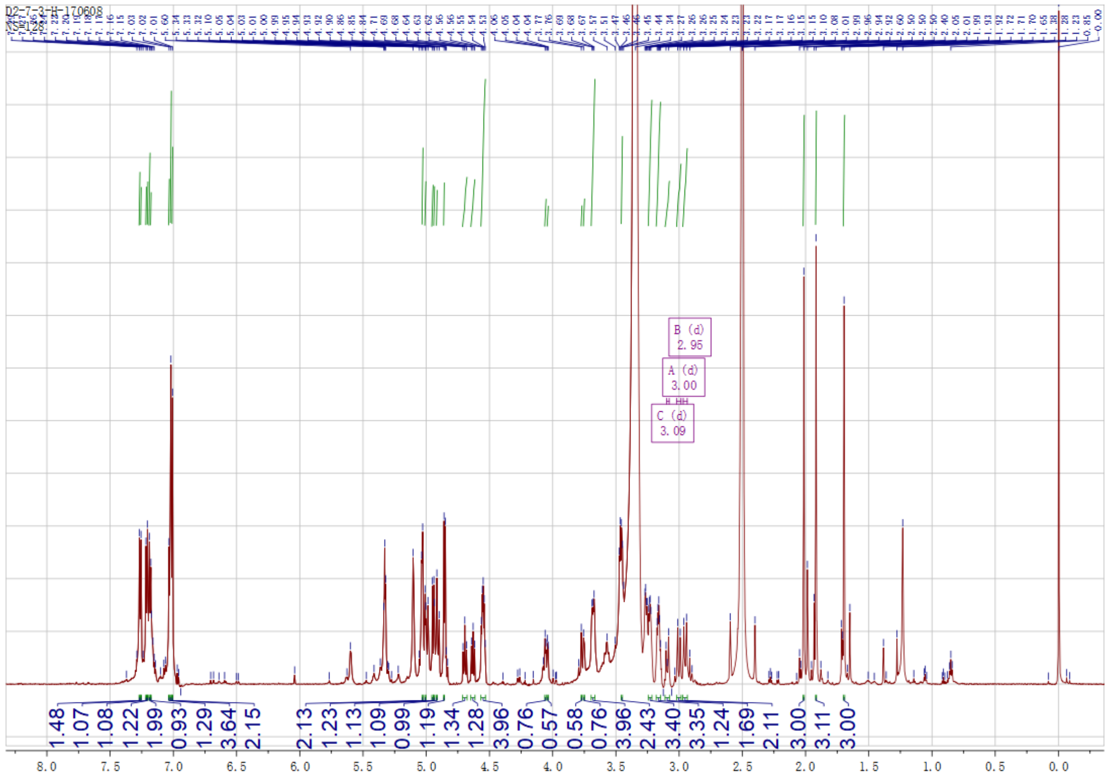


Figure S7. $^{1}$H-NMR spectrum (700 MHz, DMSO-$d_6$) of Arundinoside I (**5**).

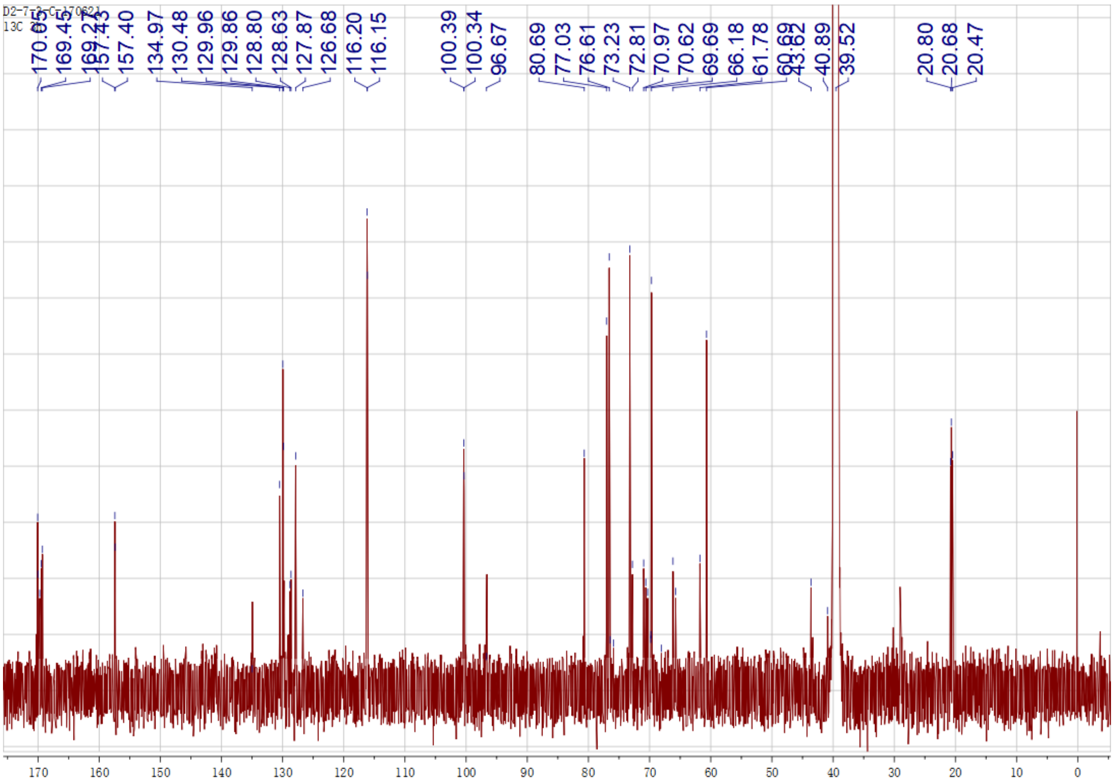


Figure S8. $^{13}$C-NMR spectrum (175 MHz, DMSO-$d_6$) of Arundinoside I (**5**).

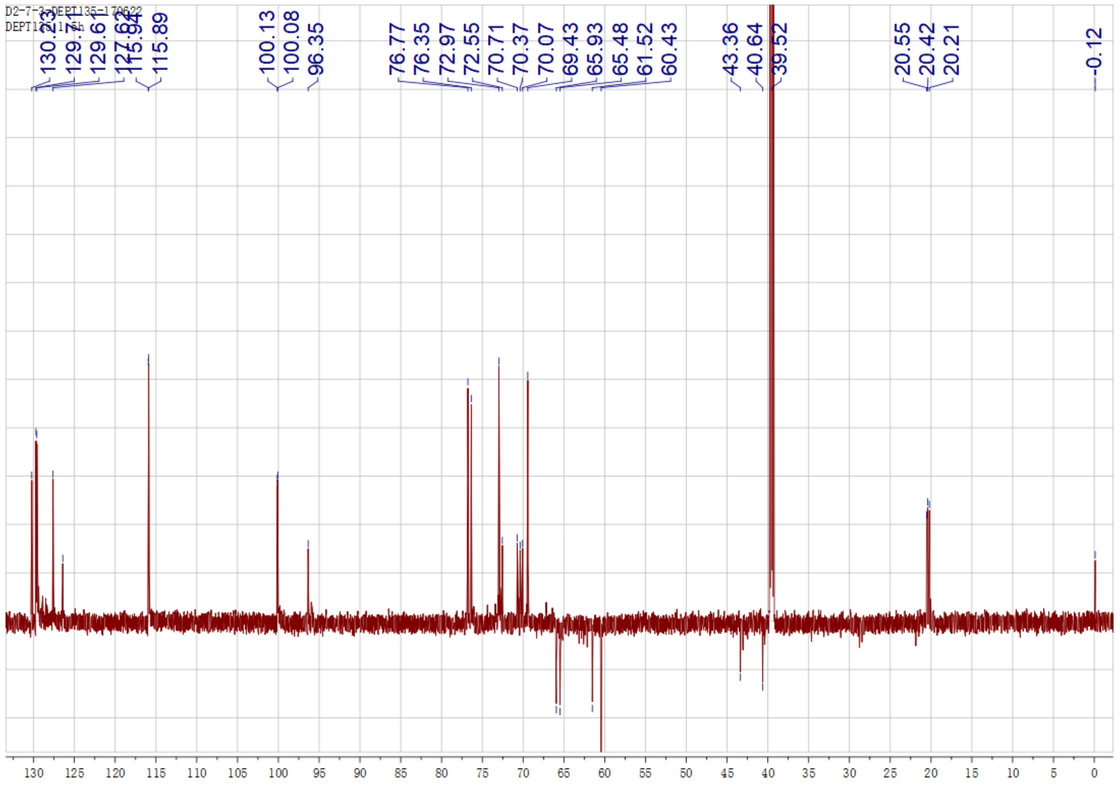


Figure S9. DEPT135 spectrum (175 MHz, DMSO-$d_6$) of Arundinoside I (**5**).

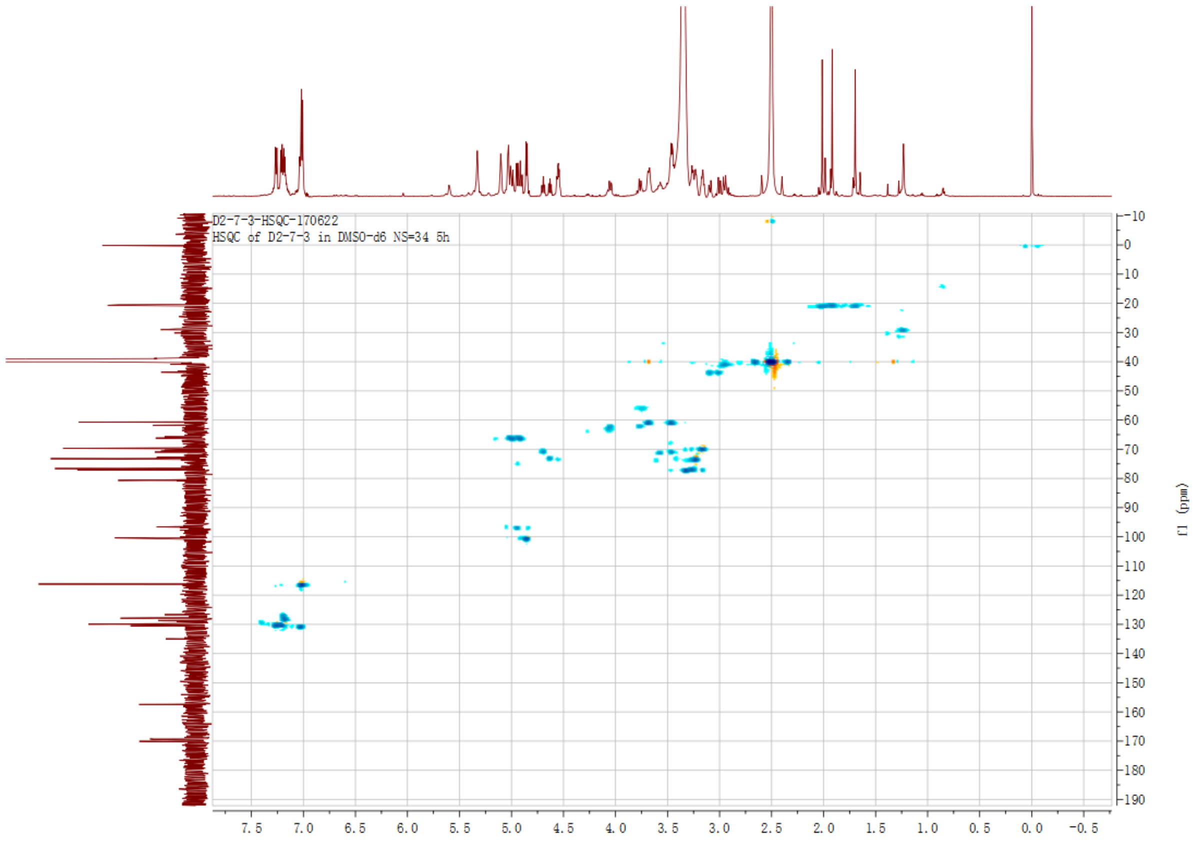


Figure S10. HSQC spectrum of Arundinoside I (**5**).

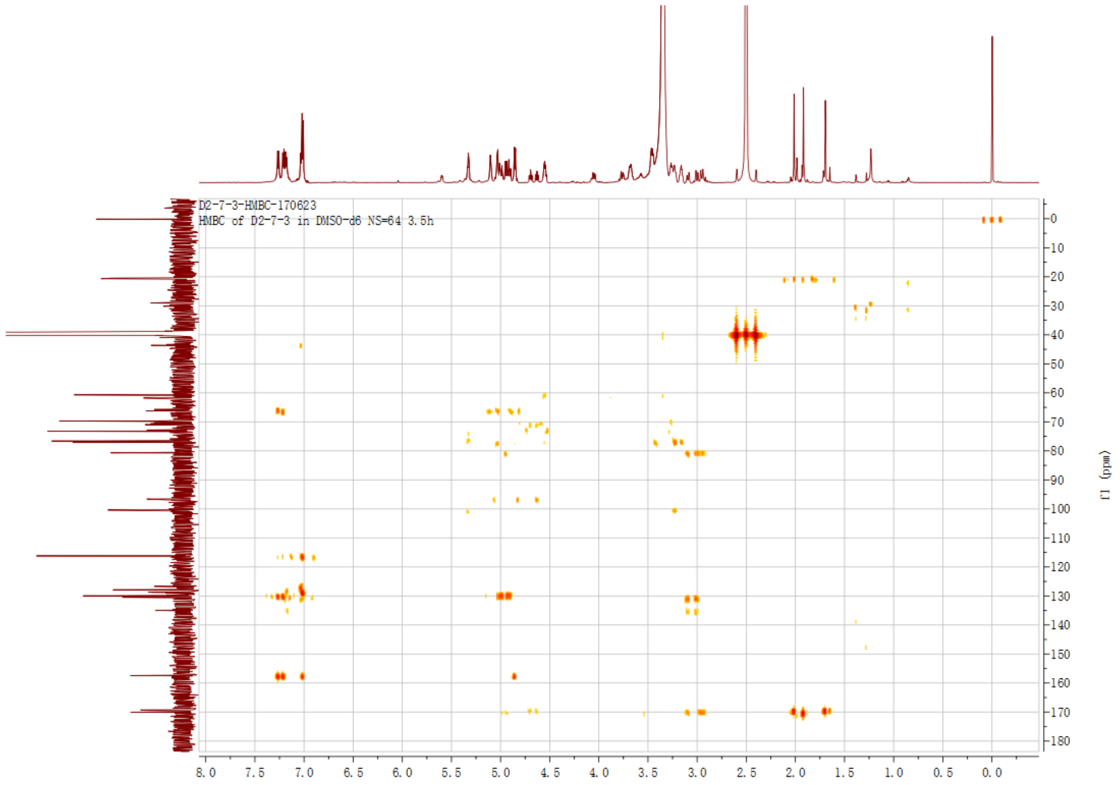


Figure S11. HMBC spectrum of Arundinoside I (**5**).

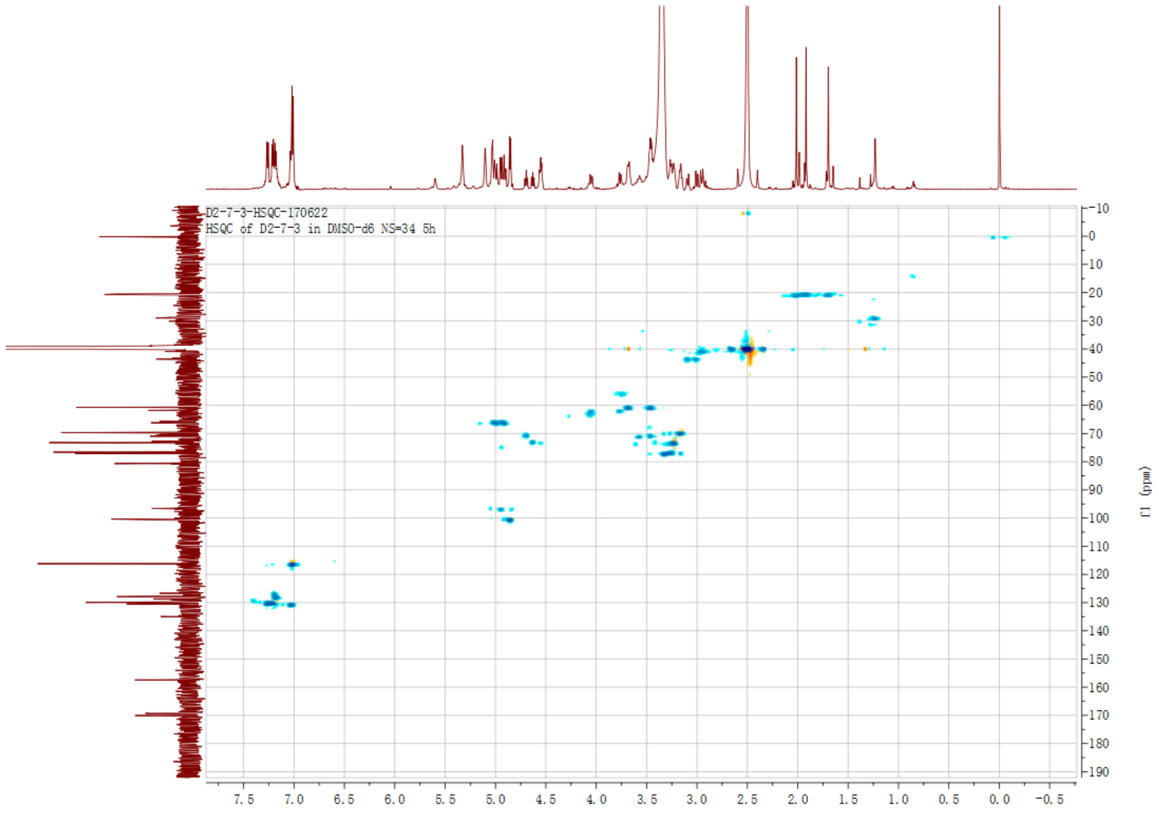


Figure S12. $^{1}H$-$^{1}H$ COSY spectrum of Arundinoside I (**5**).

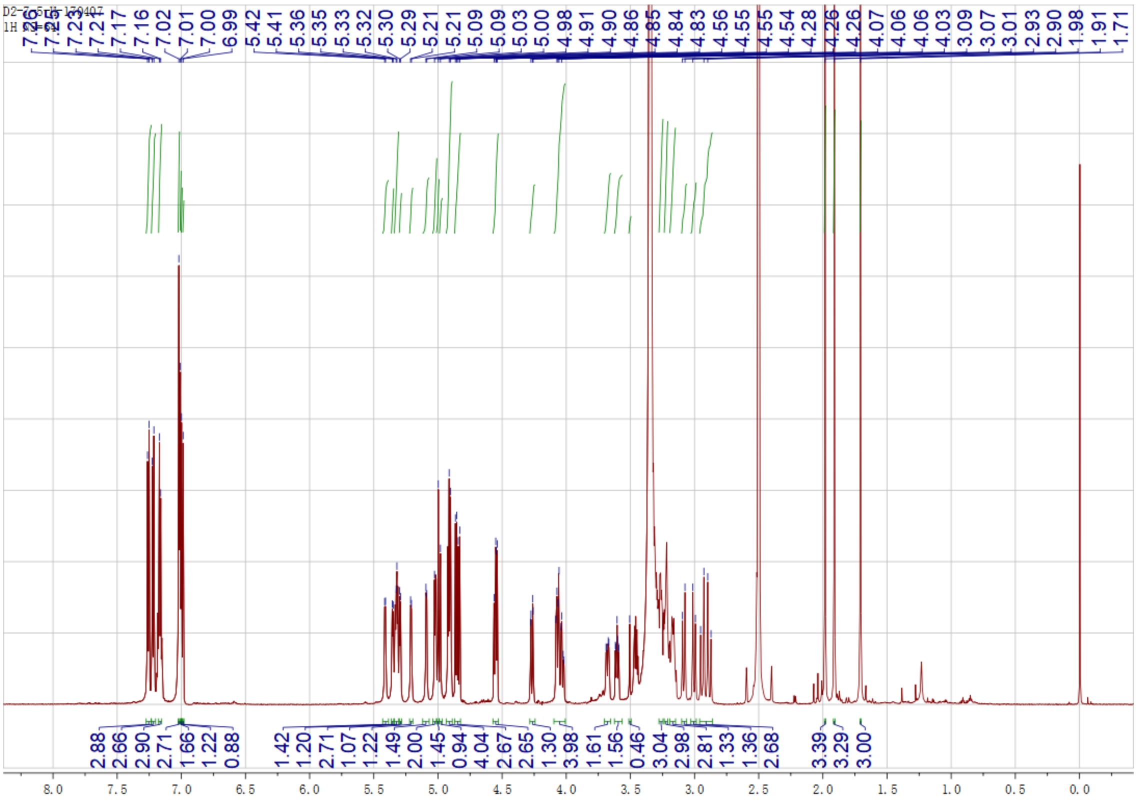

Figure S13. $^{1}$H-NMR spectrum (700 MHz, DMSO-$d_6$) of Arundinoside J (**6**).

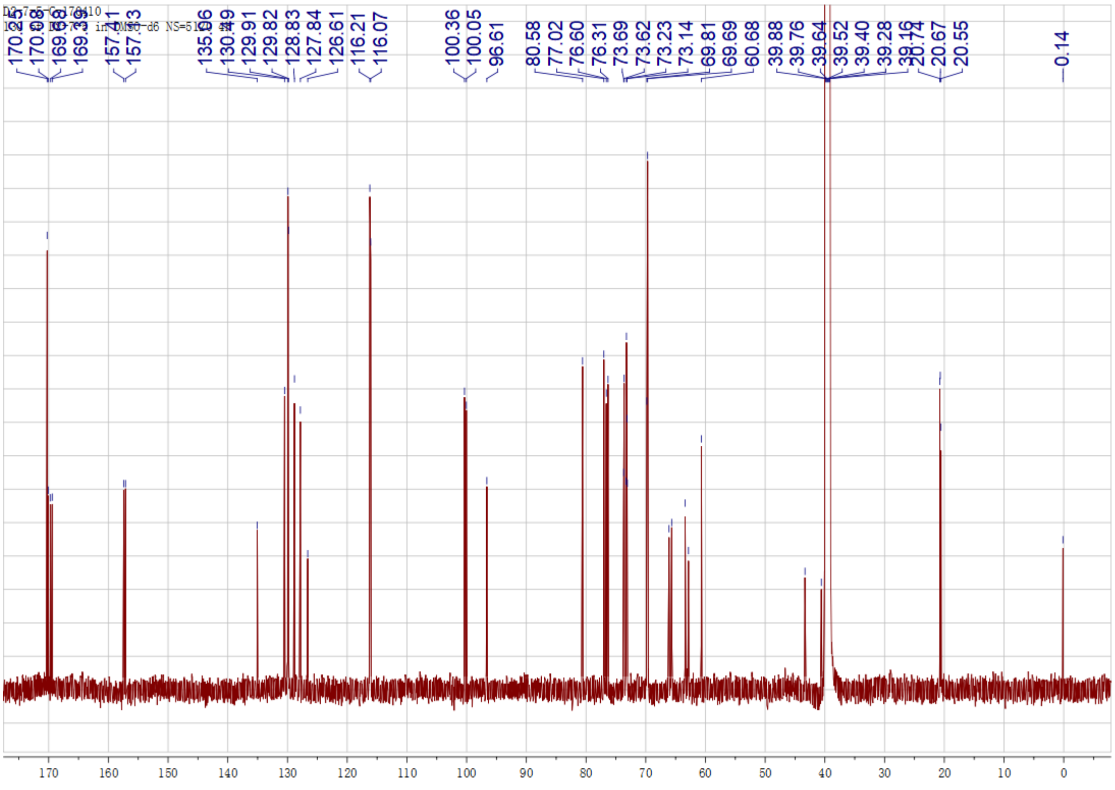

Figure S14. $^{13}$C-NMR spectrum (175 MHz, DMSO-$d_6$) of Arundinoside J (**6**).

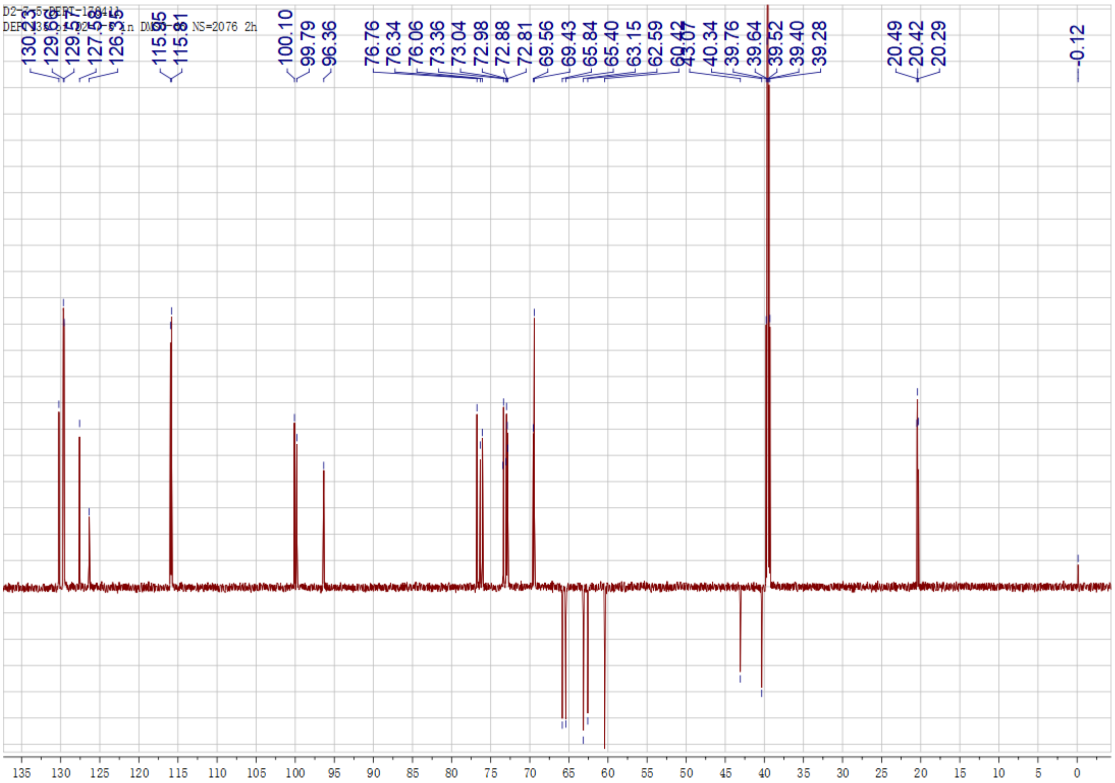


Figure S15. DEPT135 spectrum (175 MHz, DMSO-$d_6$) of Arundinoside J (**6**).

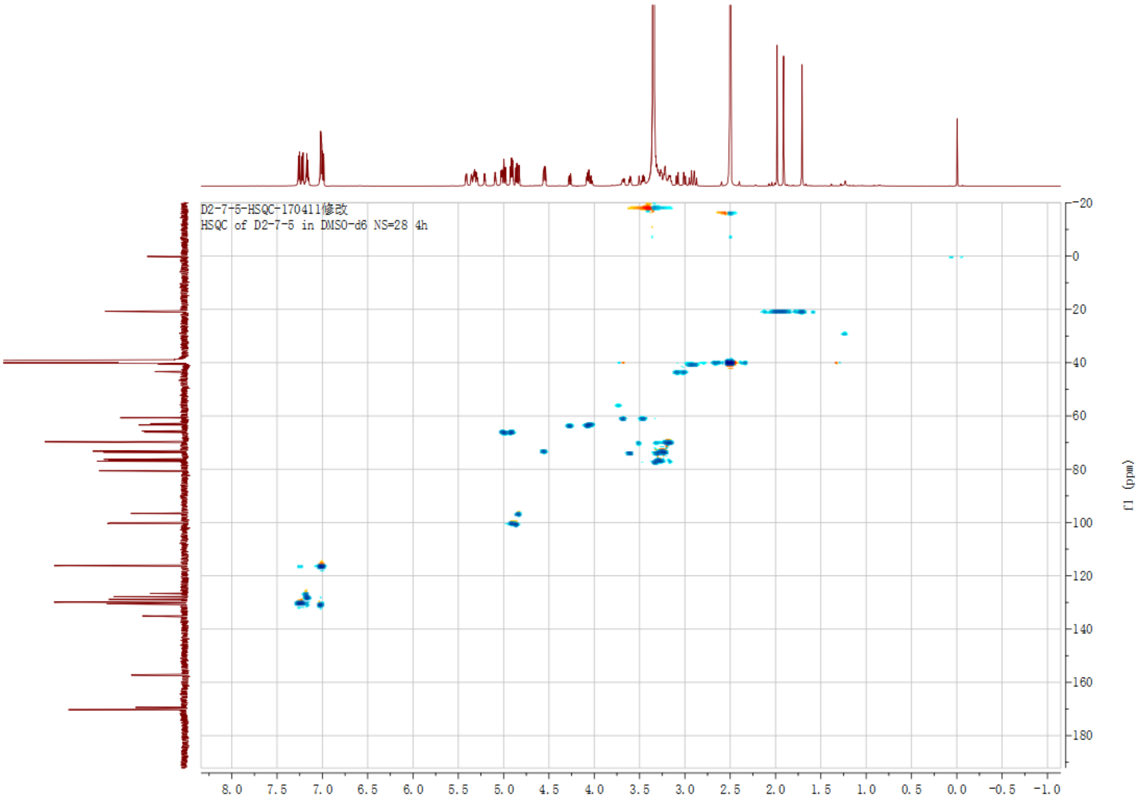


Figure S16. HSQC spectrum of Arundinoside J (**6**).

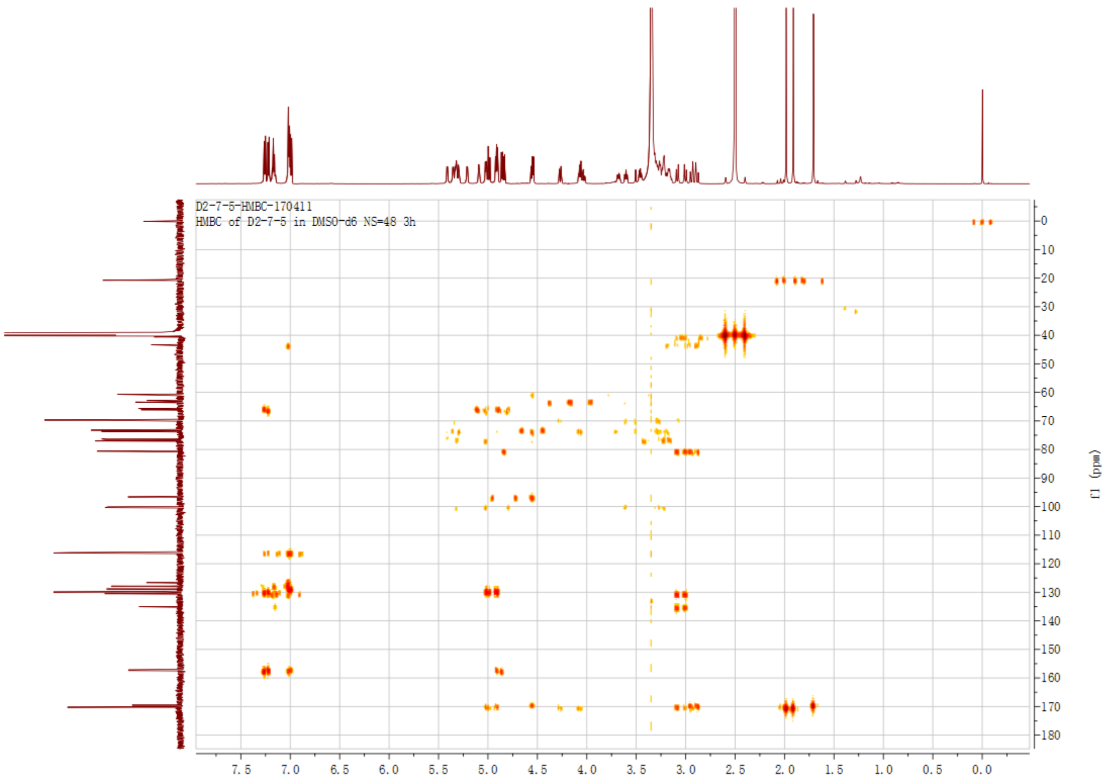


Figure S17. HMBC spectrum of Arundinoside J (**6**).

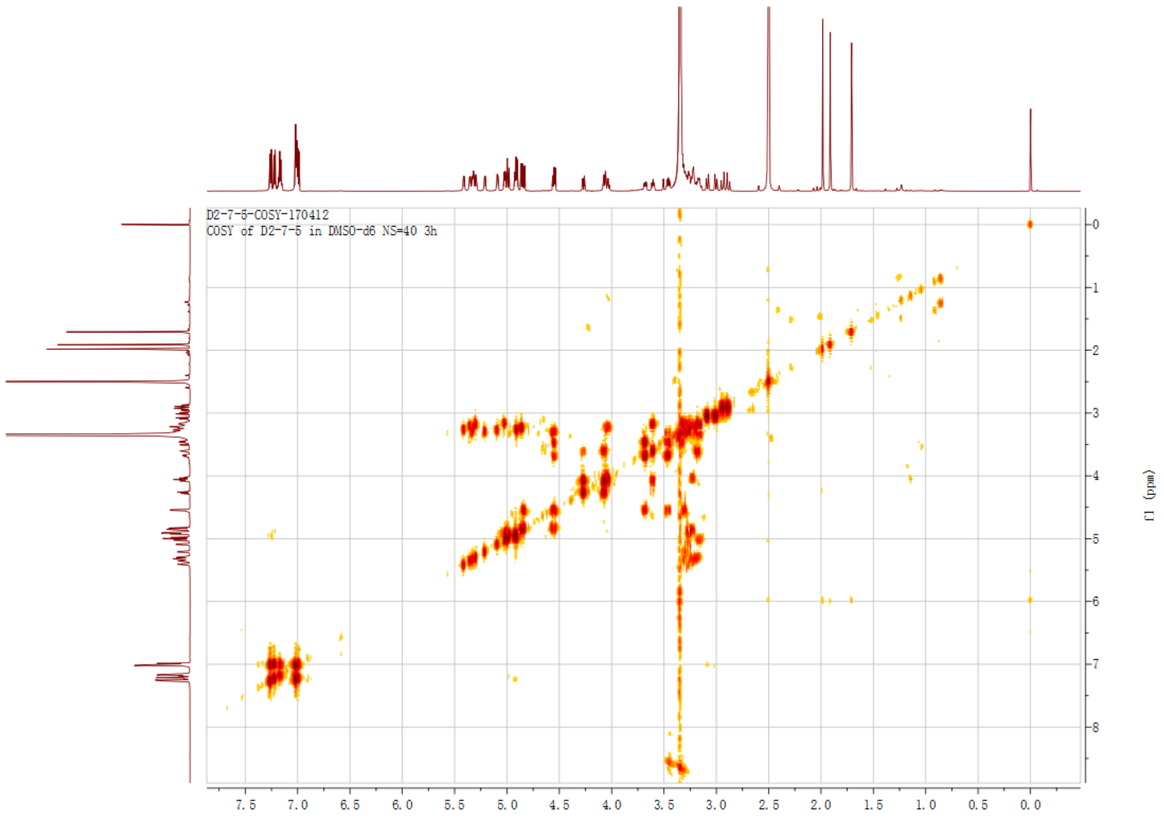


Figure S18. $^{1}H$-$^{1}H$ COSY spectrum of Arundinoside J (**6**).

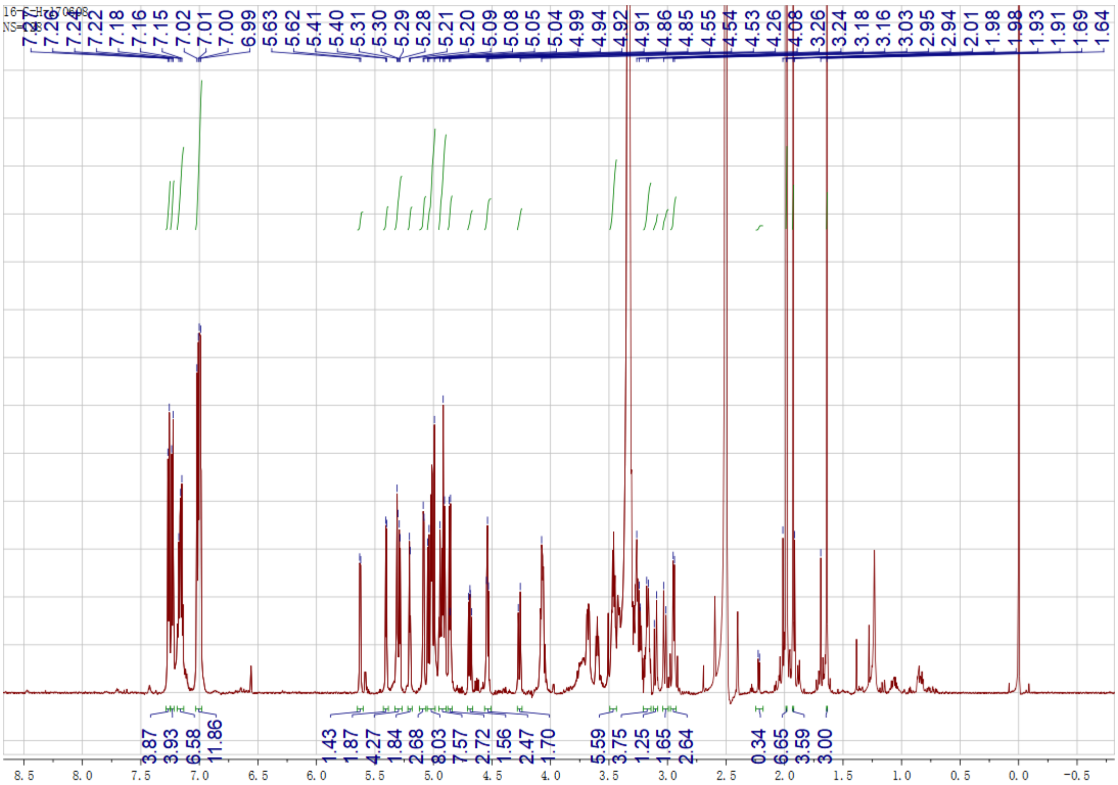

Figure S19. $^{1}$H-NMR spectrum (700 MHz, DMSO-$d_6$) of Arundinoside K (**8**).

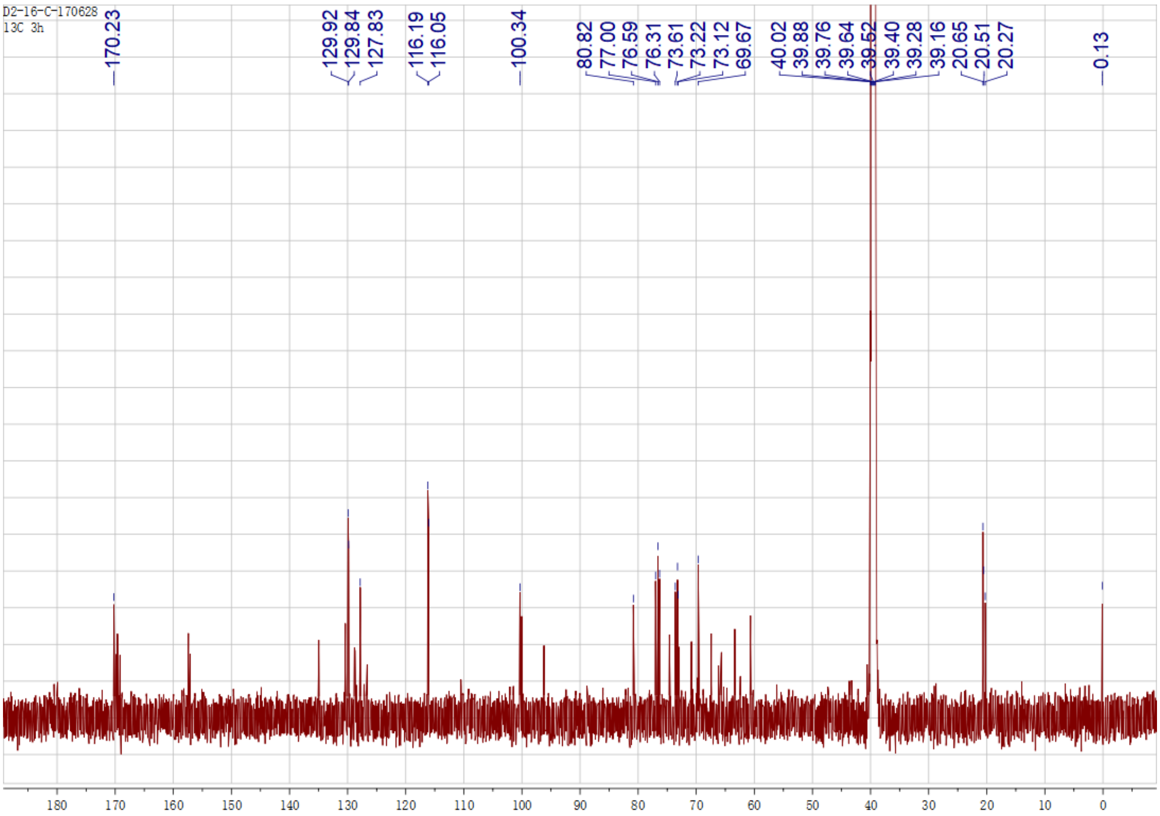

Figure S20. $^{13}$C-NMR spectrum (175 MHz, DMSO-$d_6$) of Arundinoside K (**8**).

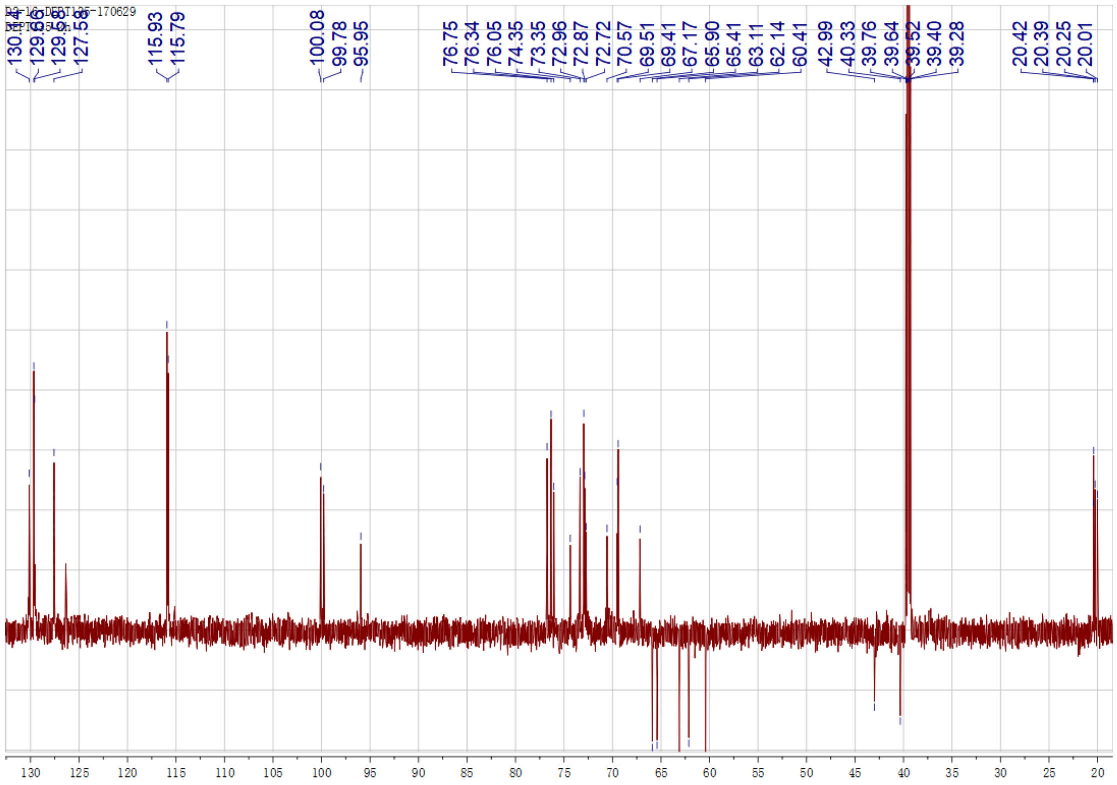

Figure S21. DEPT135 spectrum (175 MHz, DMSO-$d_6$) of Arundinoside K (**8**).

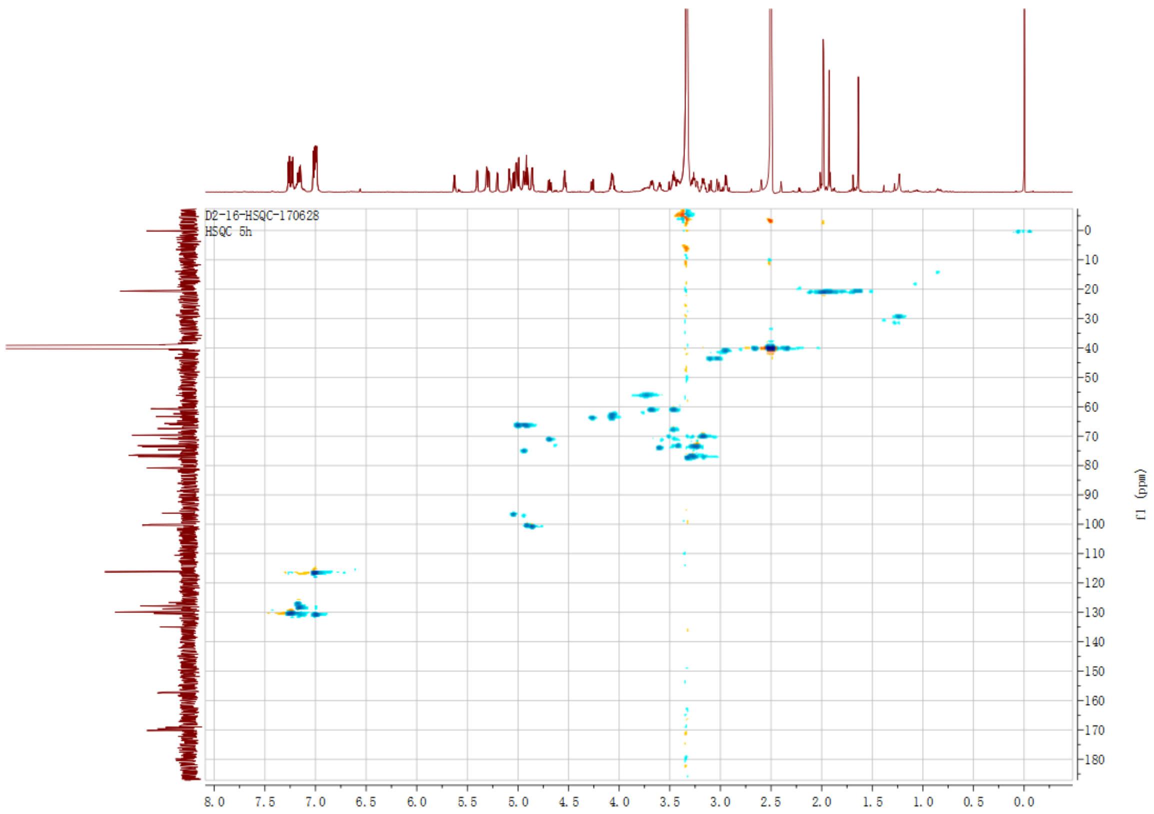

Figure S22. HSQC spectrum of Arundinoside K (**8**).

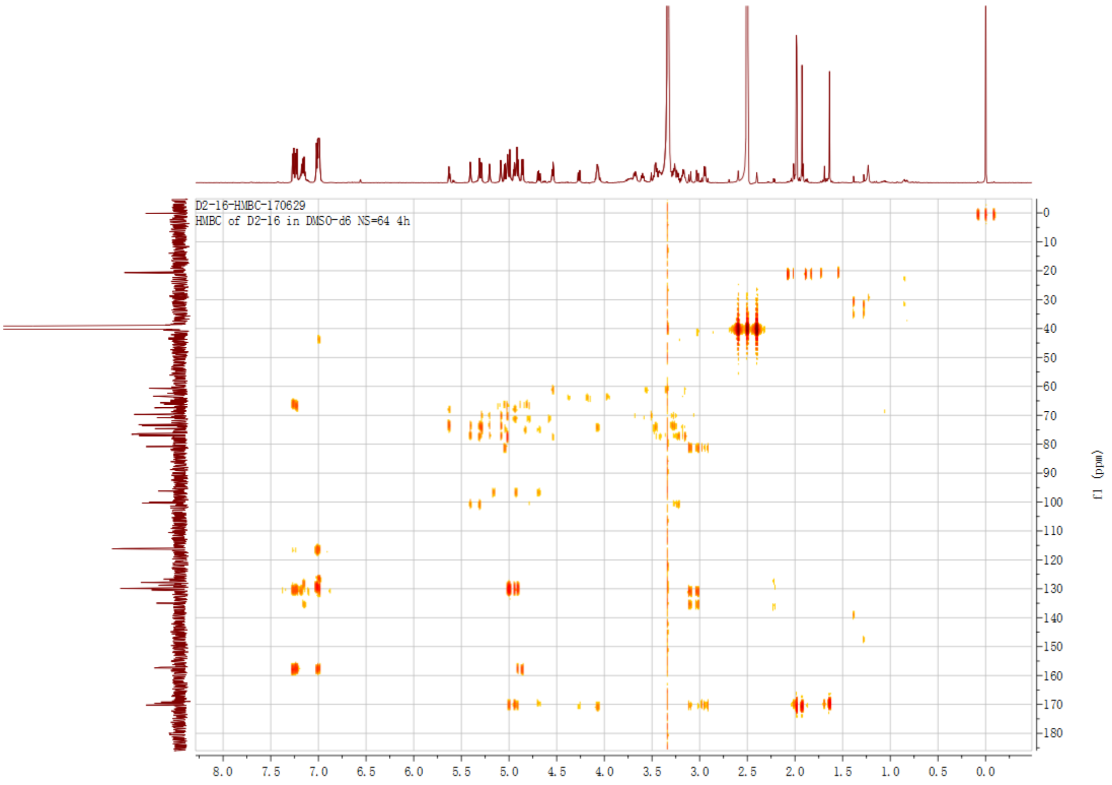


Figure S23. HMBC spectrum of Arundinoside K (**8**).

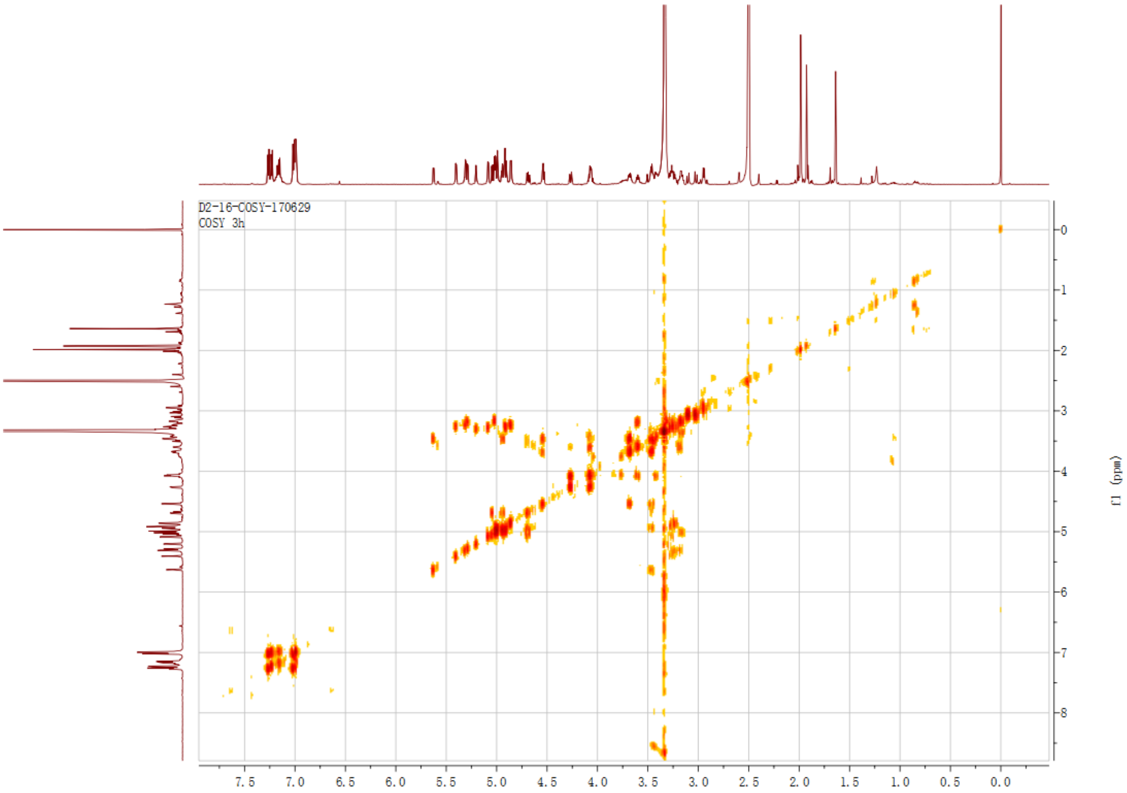


Figure S24. $^{1}$H-$^{1}$H COSY spectrum of Arundinoside K (**8**).

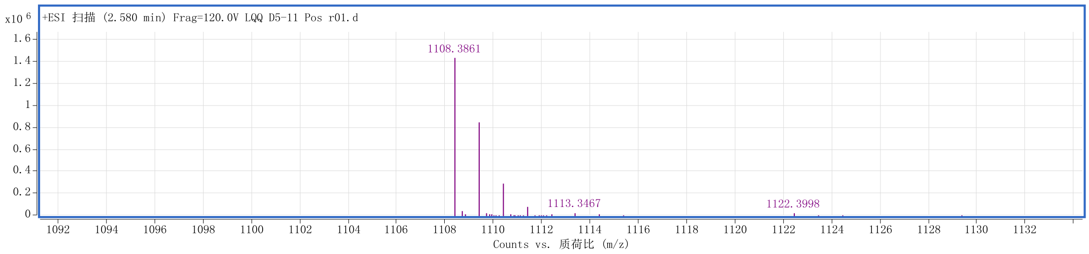


Figure S25. HRESIMS spectrum of Arundinoside D (**1**).

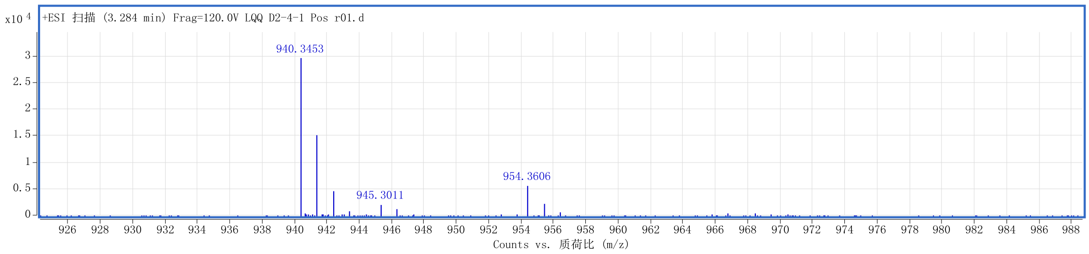


Figure S26. HRESIMS spectrum of Arundinoside H (**2**).

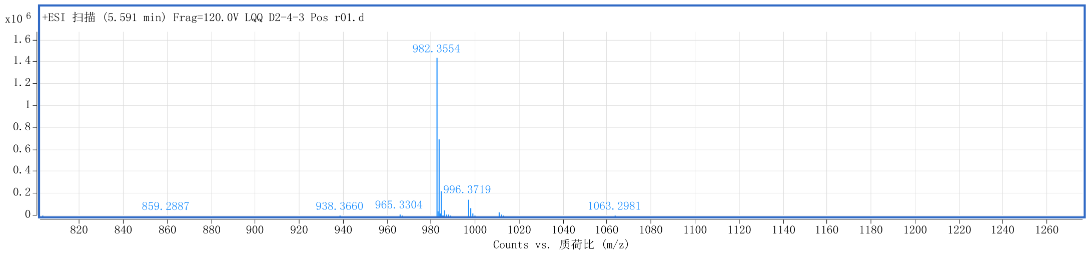


Figure S27. HRESIMS spectrum of Arundinoside G (**3**).

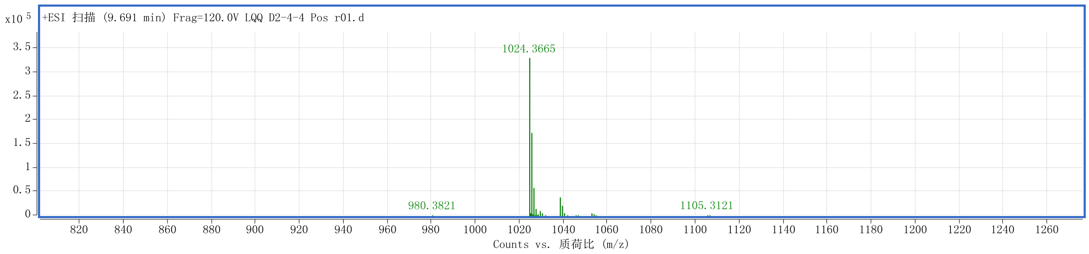


Figure S28. HRESIMS spectrum of Arundinoside F (**4**).

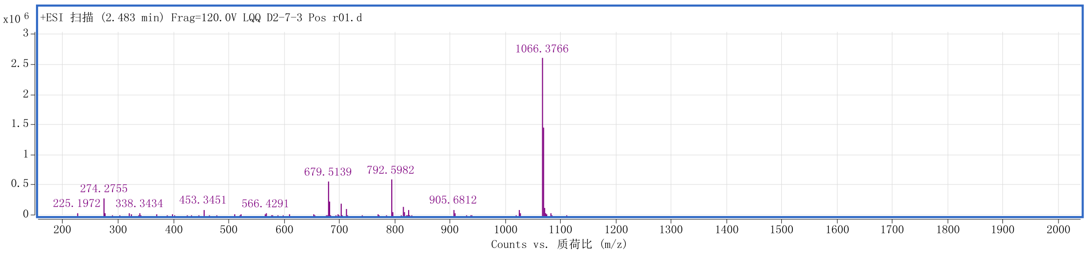


Figure S29. HRESIMS spectrum of Arundinoside I (**5**).

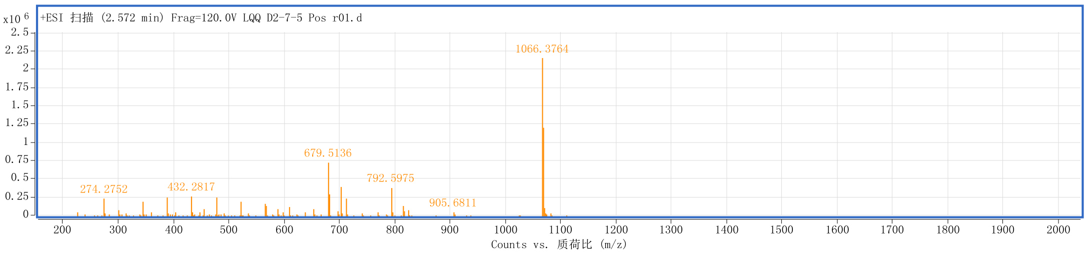


Figure S30. HRESIMS spectrum of Arundinoside J (**6**).

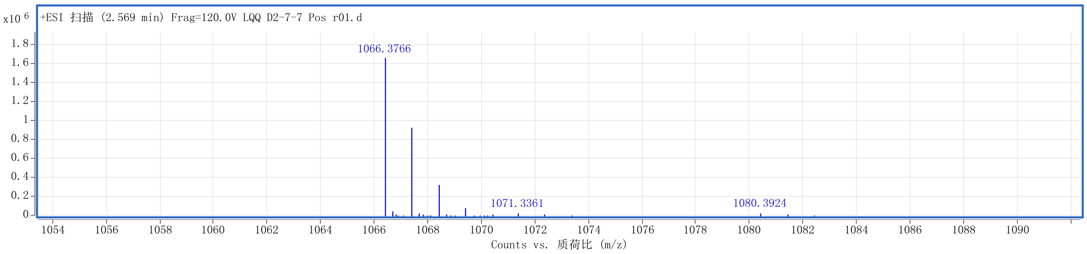


Figure S31. HRESIMS spectrum of Arundinoside E (**7**).

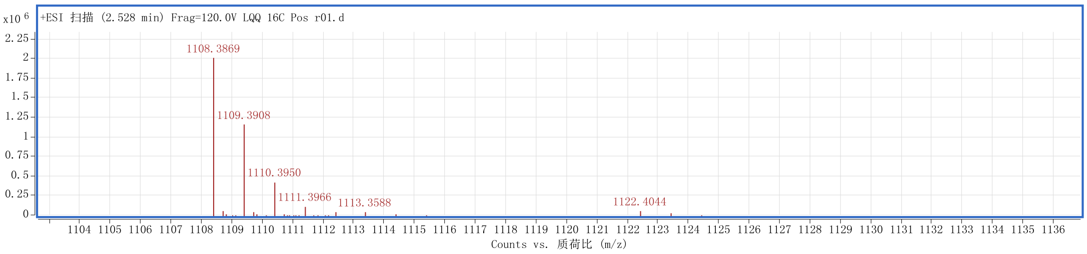


Figure S32. HRESIMS spectrum of Arundinoside K (**8**).

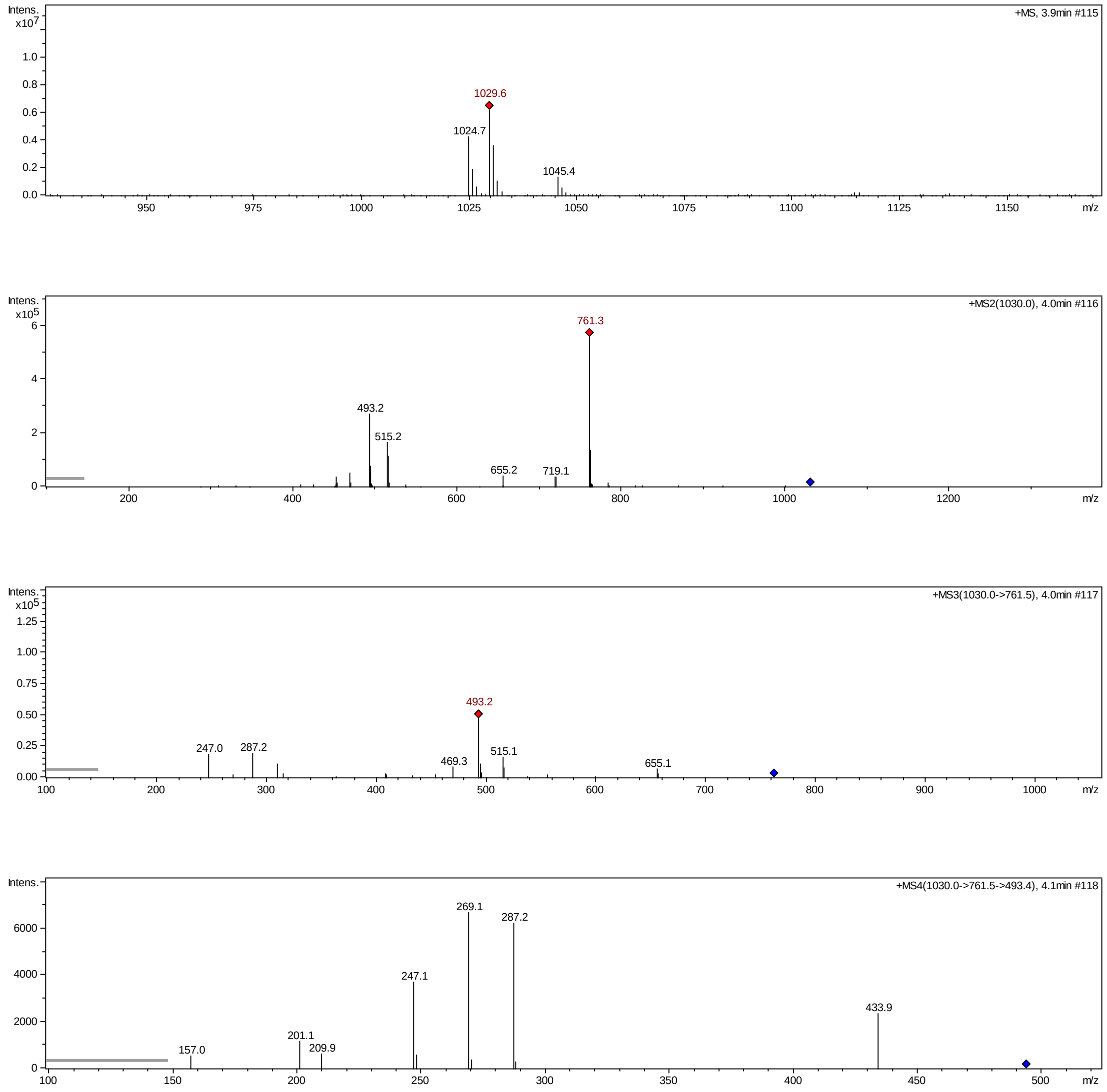


Figure S33. HPLC-ESI-MS$^n$ spectrum of peak **A1**.

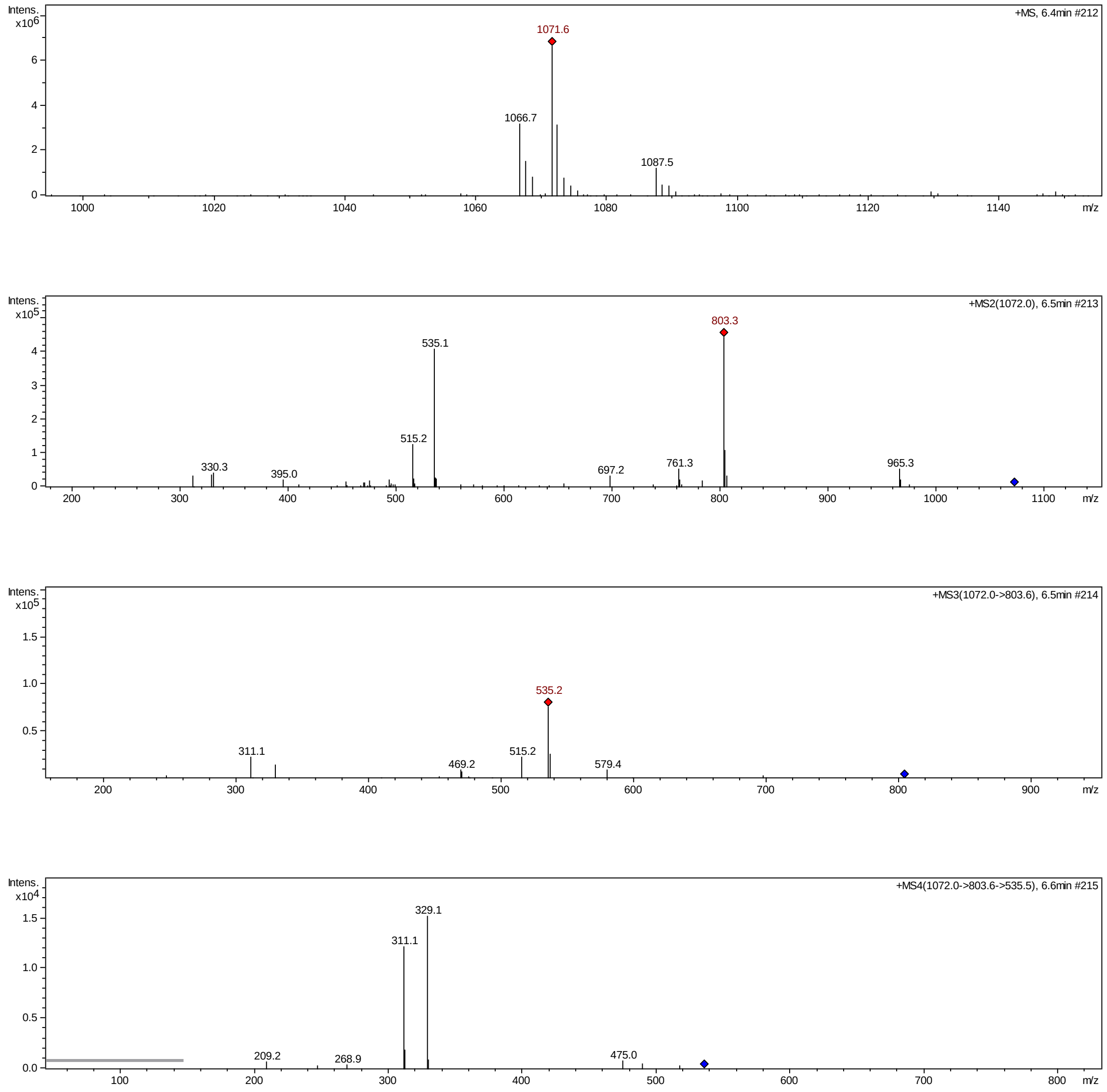


Figure S34. HPLC-ESI-MS$^n$ spectrum of peak **A2**.

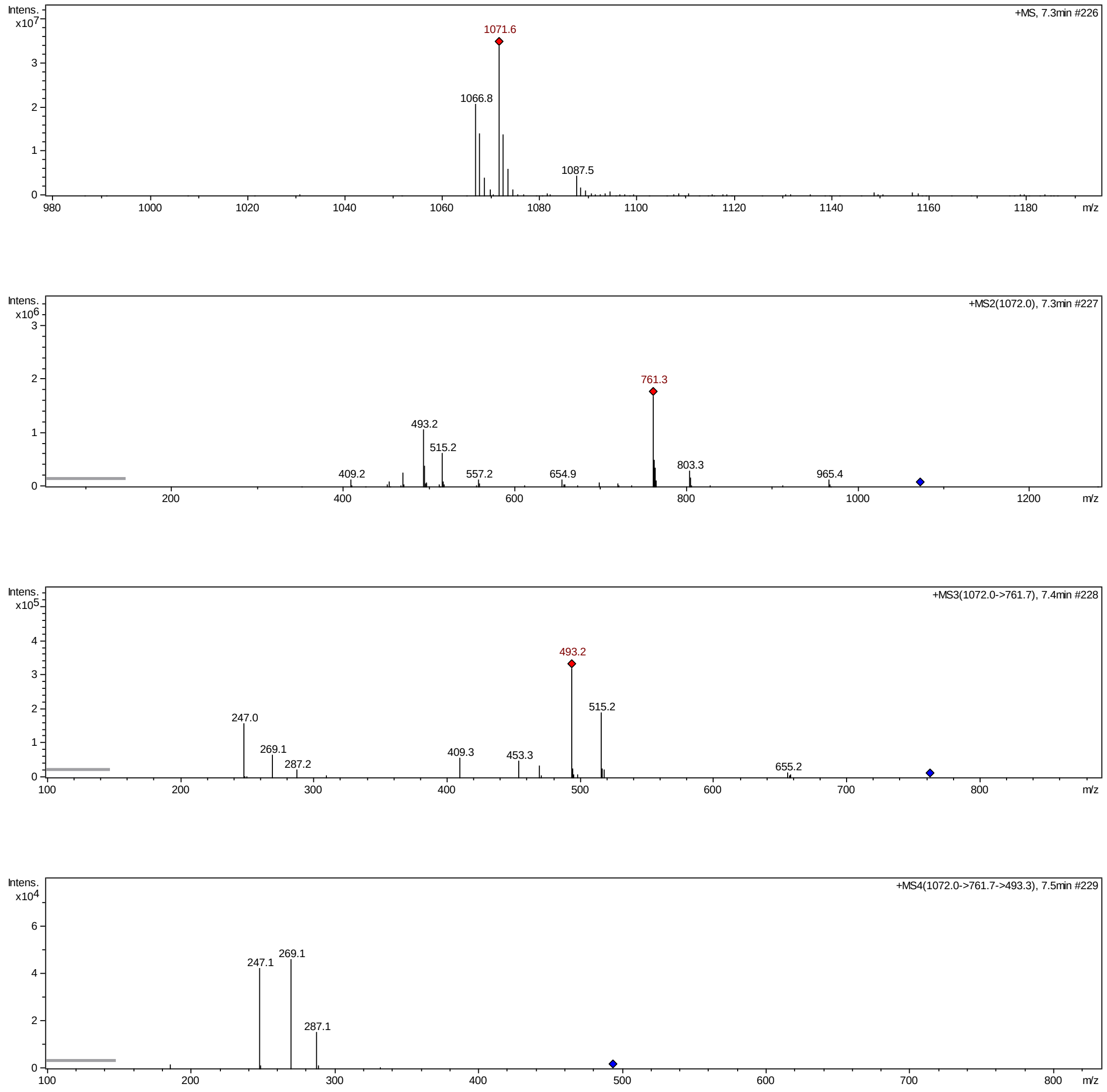


Figure S35. HPLC-ESI-MS$^n$ spectrum of peak **A3**.

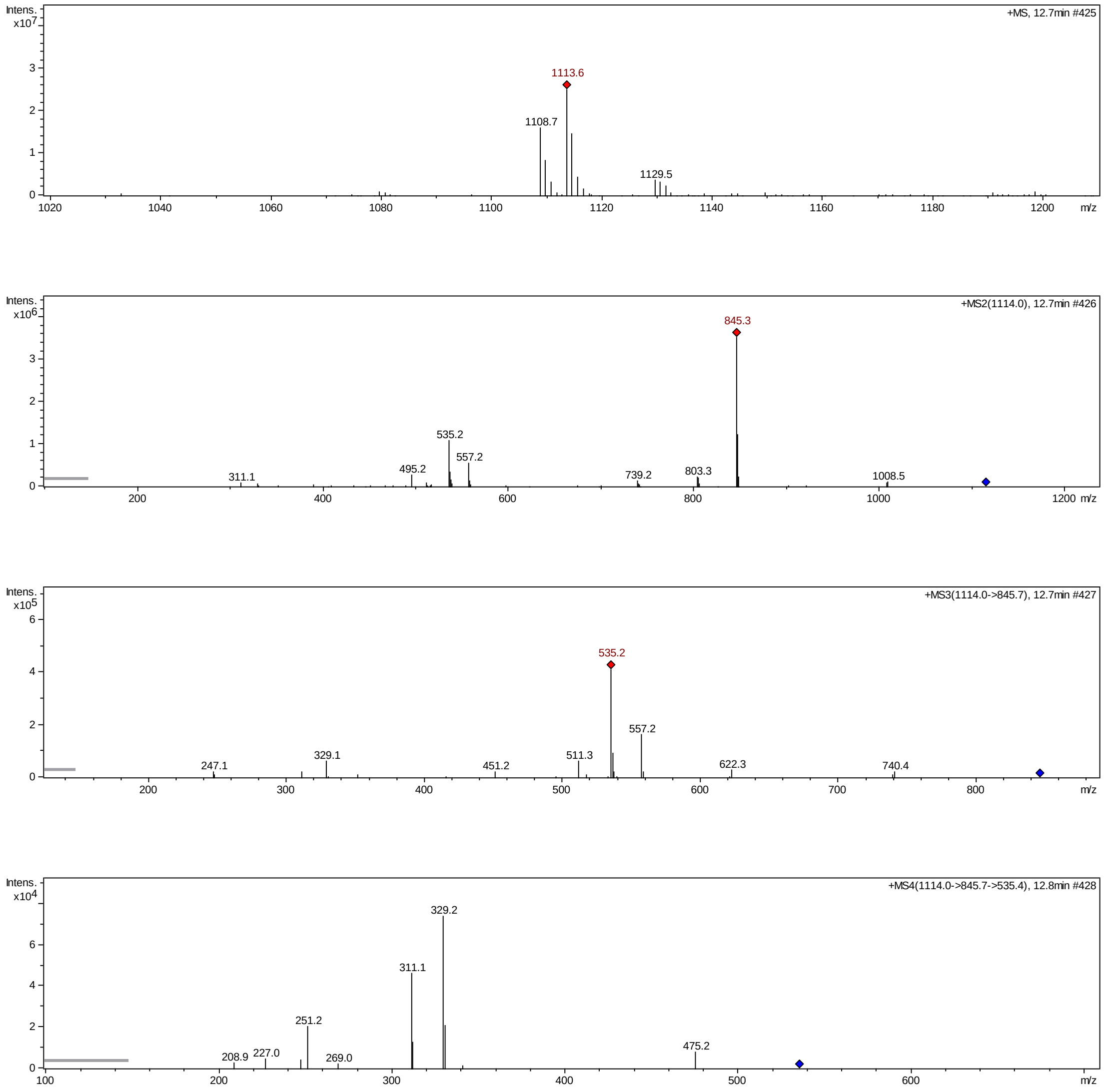


Figure S36. HPLC-ESI-MS$^n$ spectrum of peak **A4**.

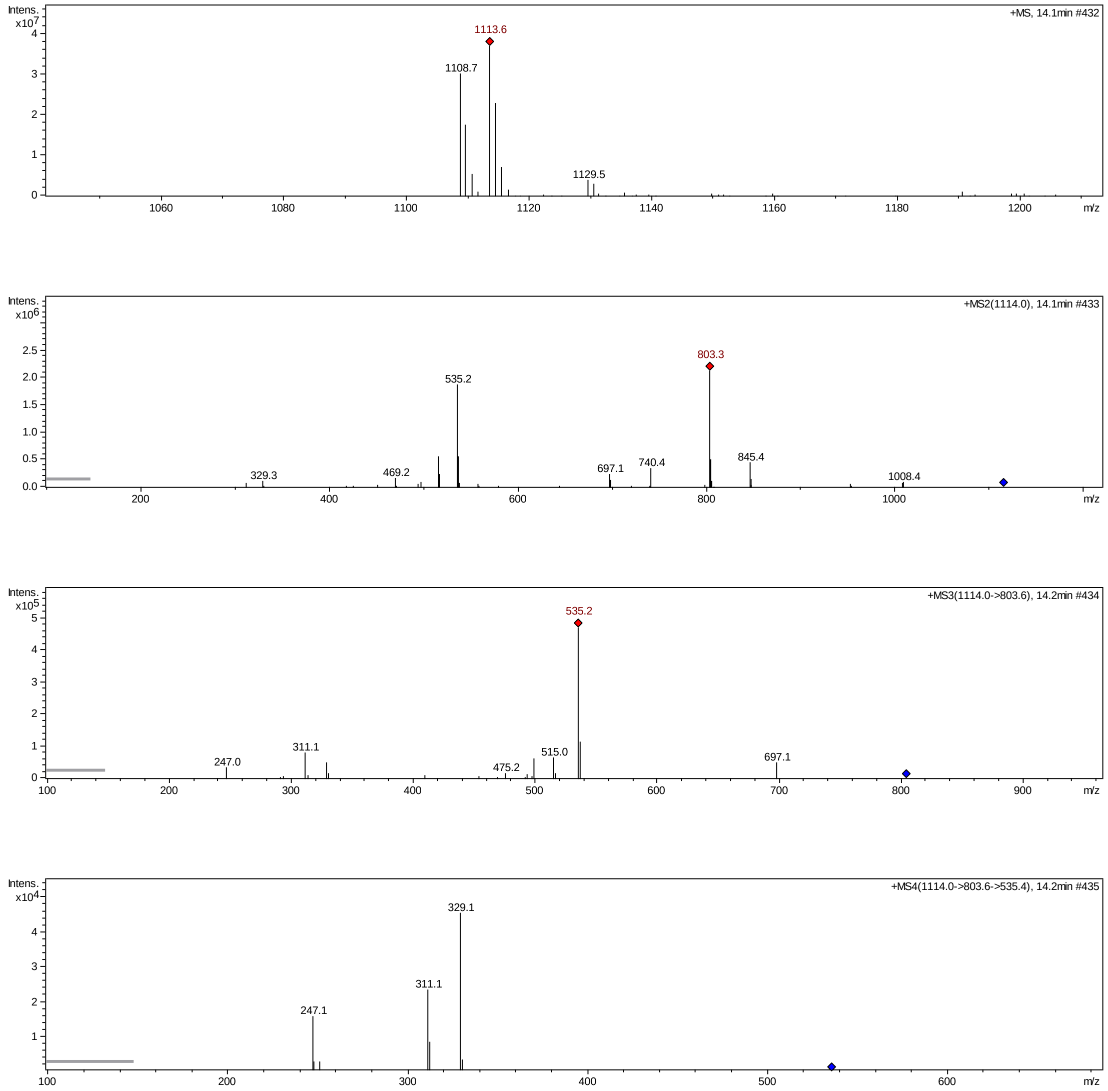


Figure S37. HPLC-ESI-MS$^n$ spectrum of peak **A5**.

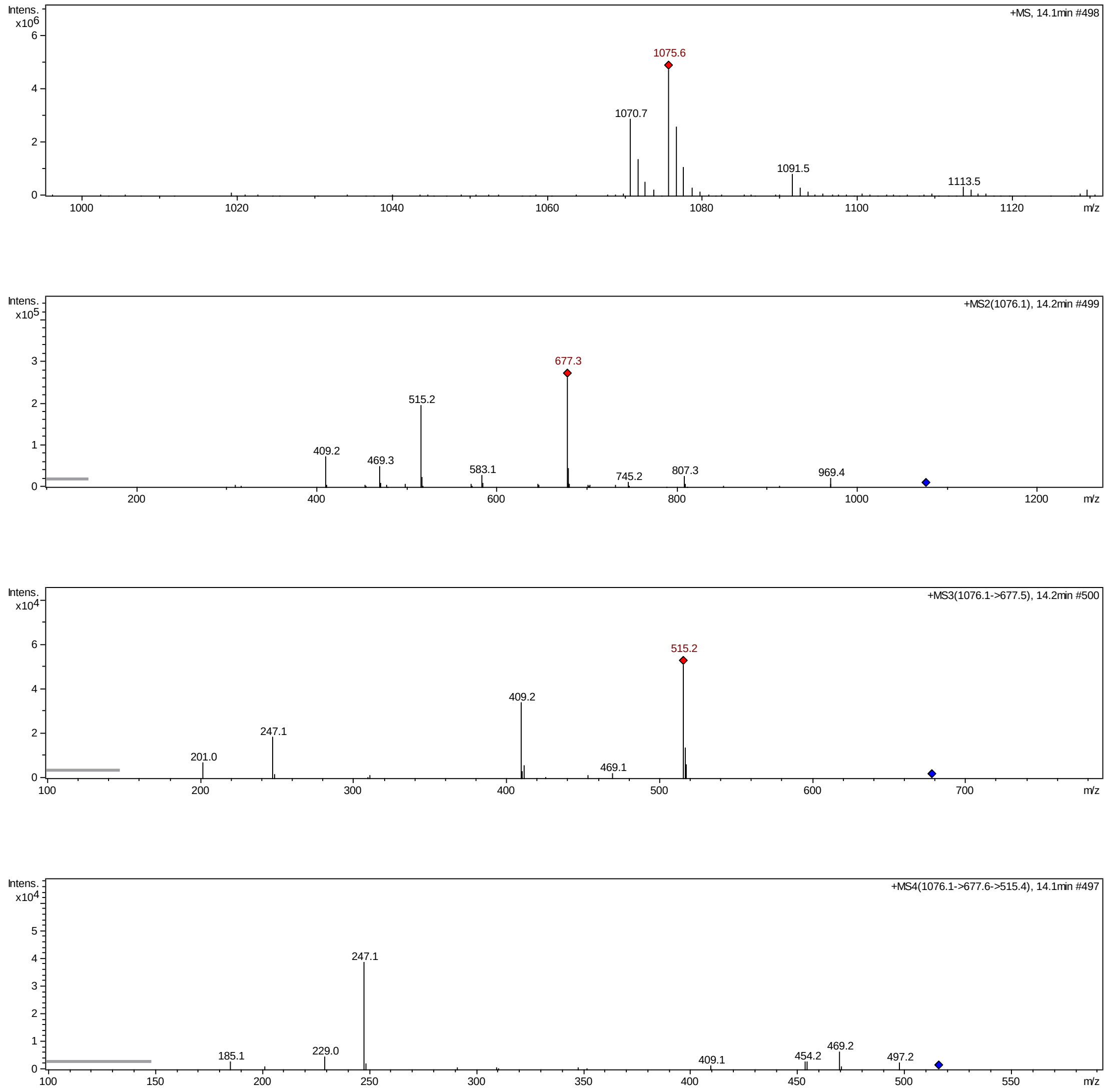


Figure S38. HPLC-ESI-MS$^n$ spectrum of peak **A6**.

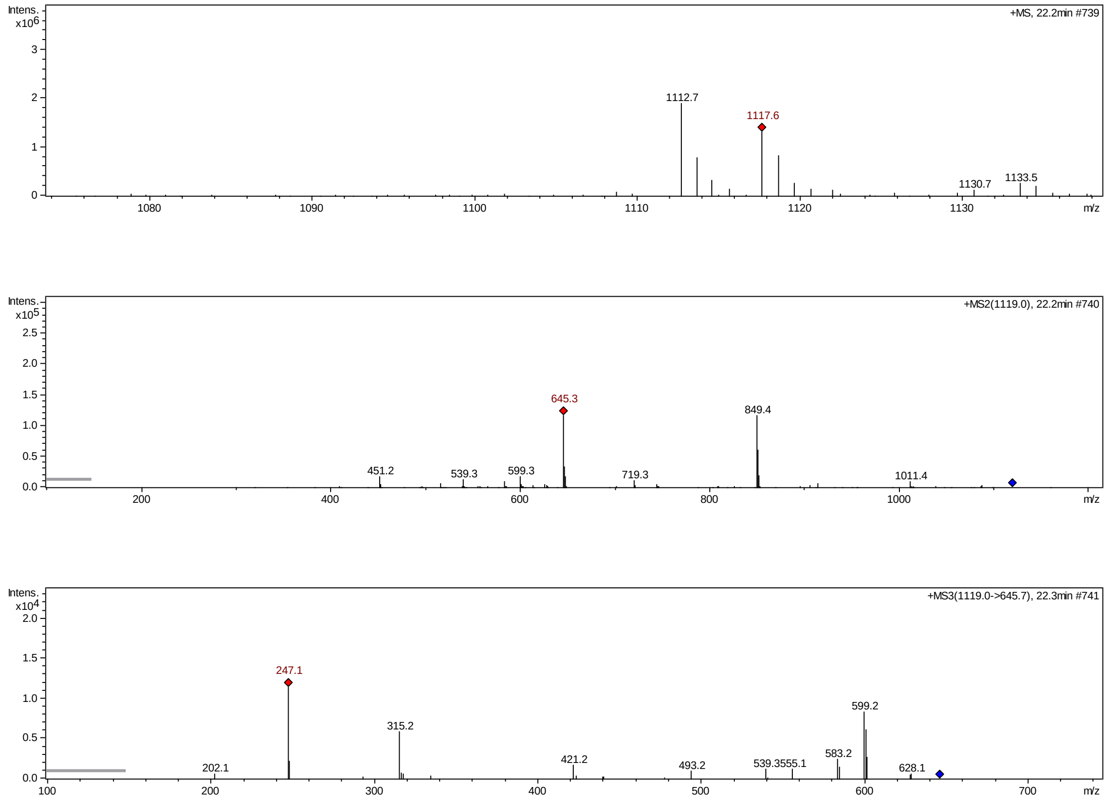


Figure S39. HPLC-ESI-MS$^n$ spectrum of peak **A7**.

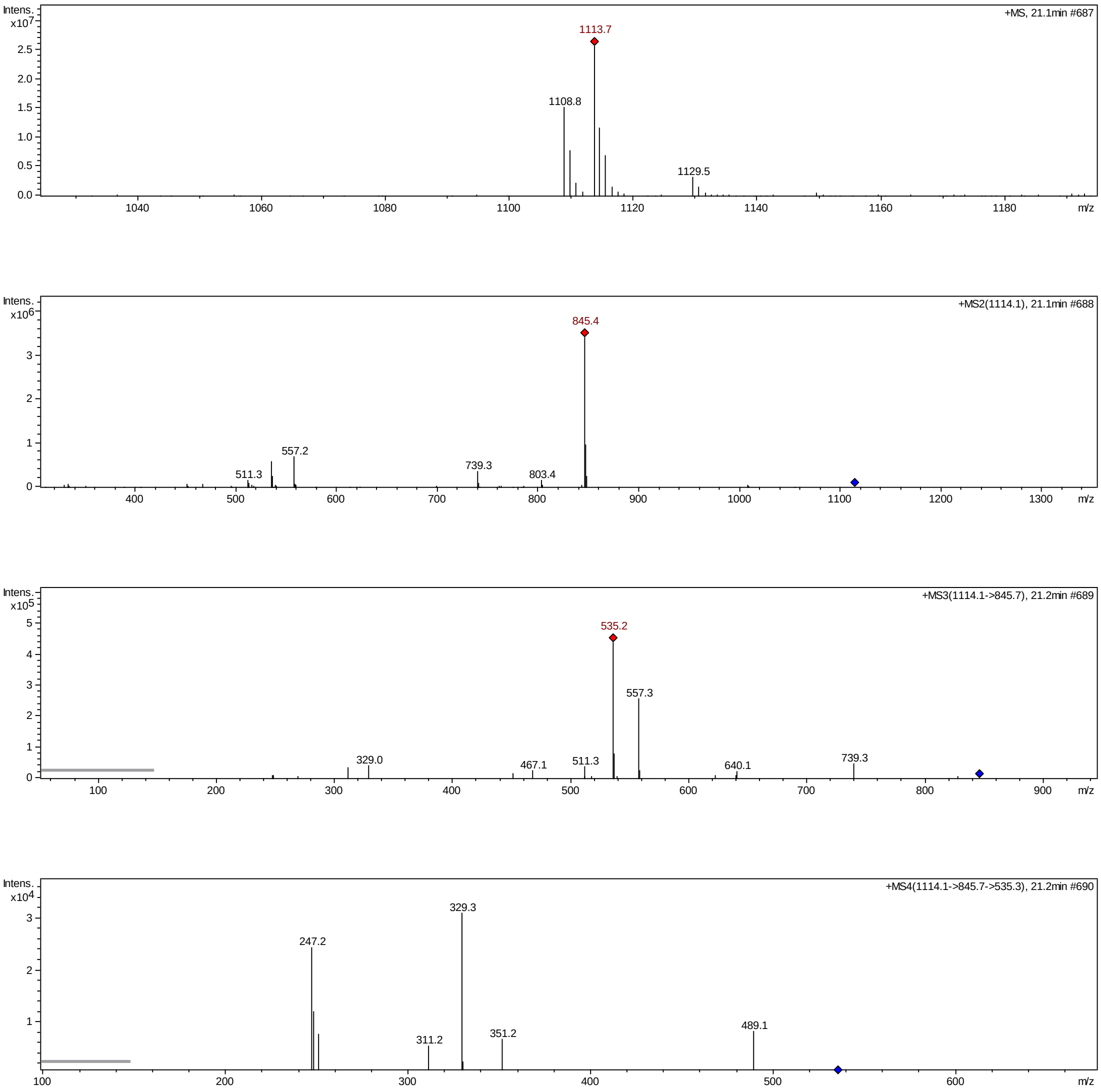


Figure S40. HPLC-ESI-MS$^n$ spectrum of peak **A8**.

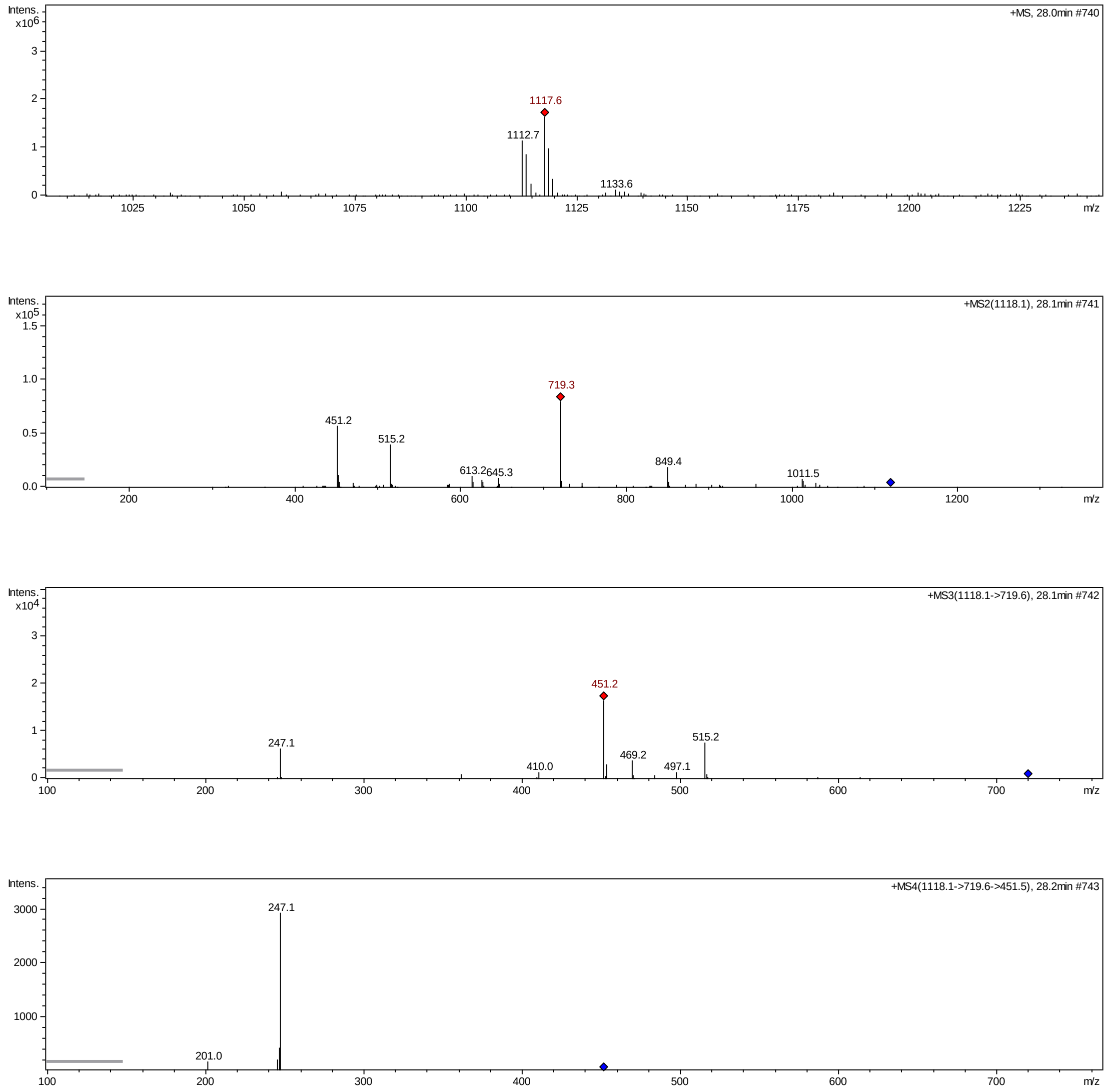


Figure S41. HPLC-ESI-MS$^n$ spectrum of peak **A9**.

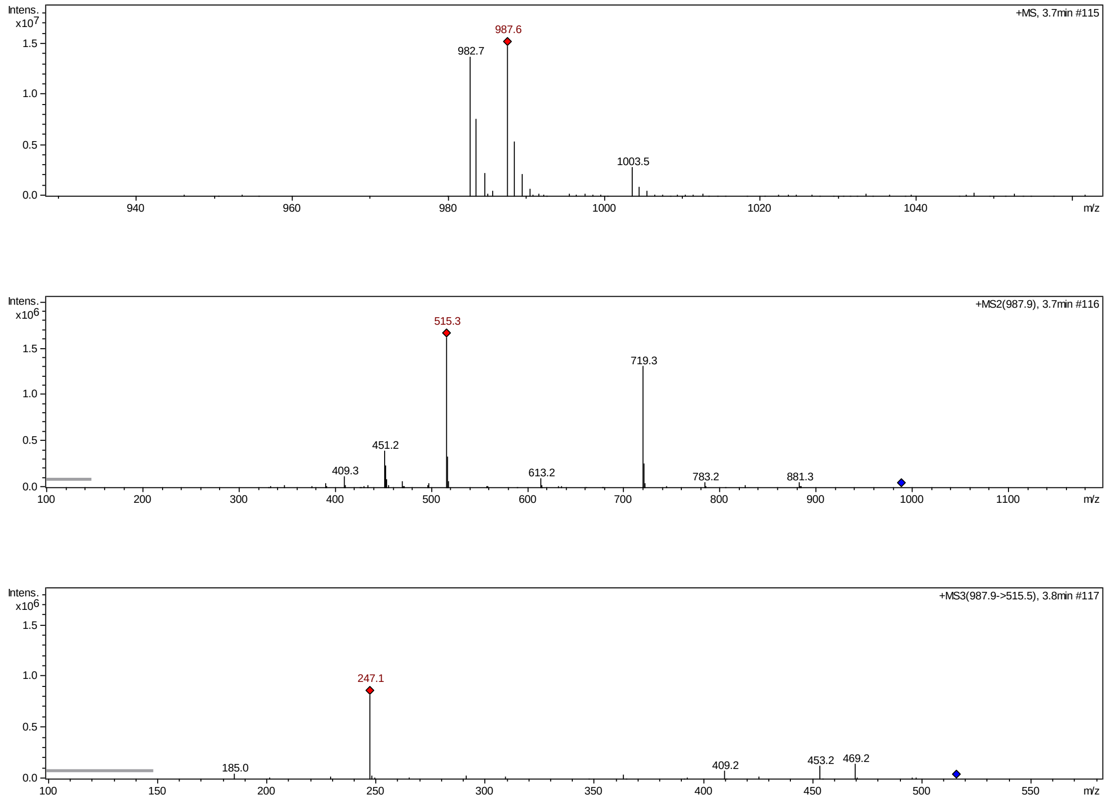


Figure S42. HPLC-ESI-MS$^n$ spectrum of peak **B1**.

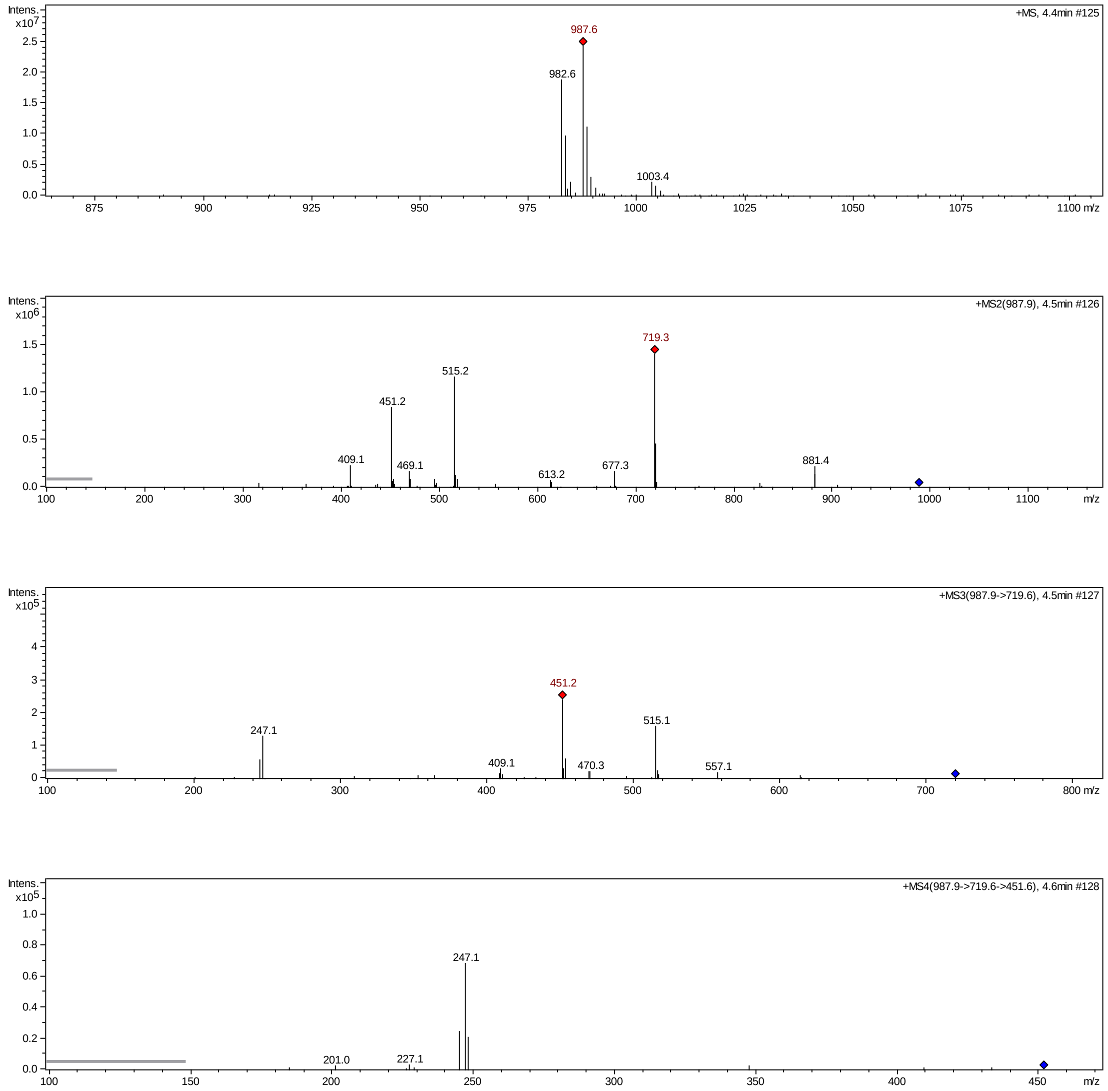


Figure S43. HPLC-ESI-MS$^n$ spectrum of peak **B2**.

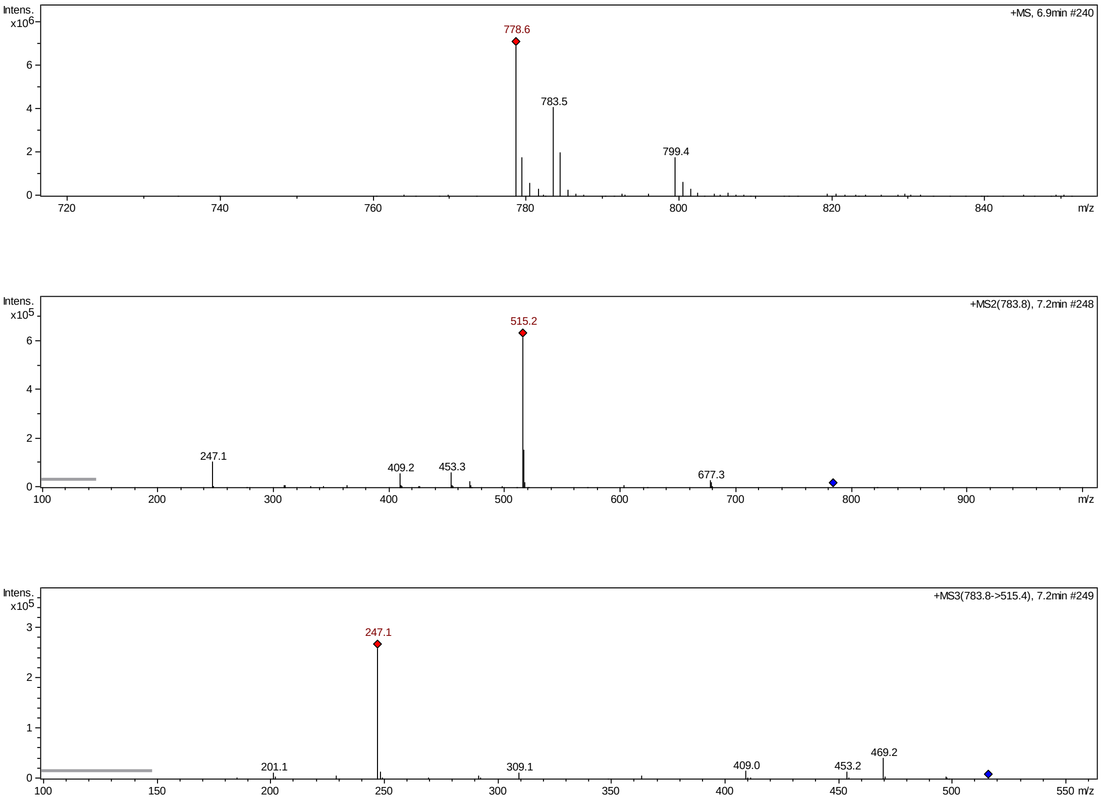


Figure S44. HPLC-ESI-MS$^n$ spectrum of peak **B3**.

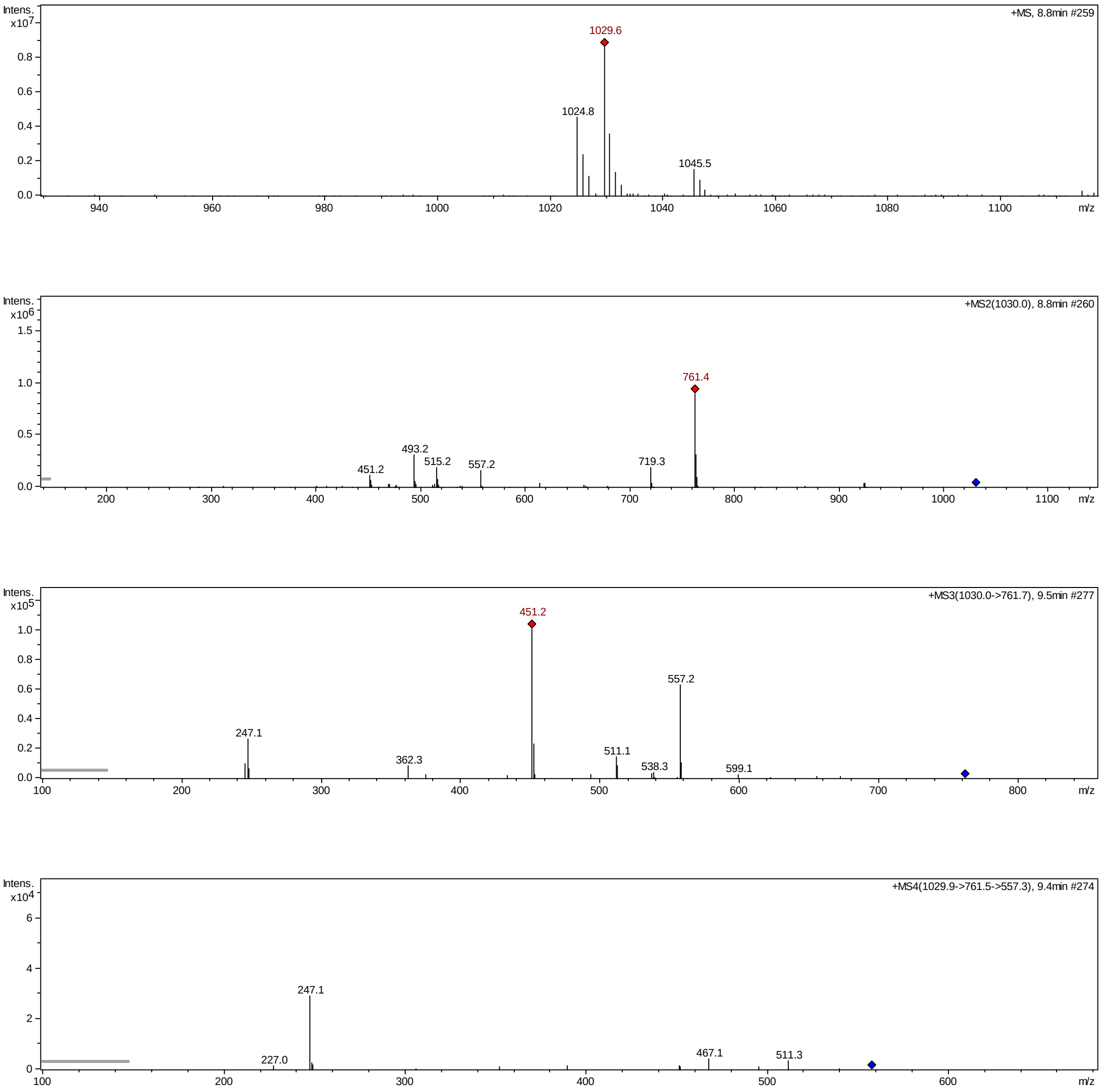


Figure S45. HPLC-ESI-MS$^n$ spectrum of peak **B4**.

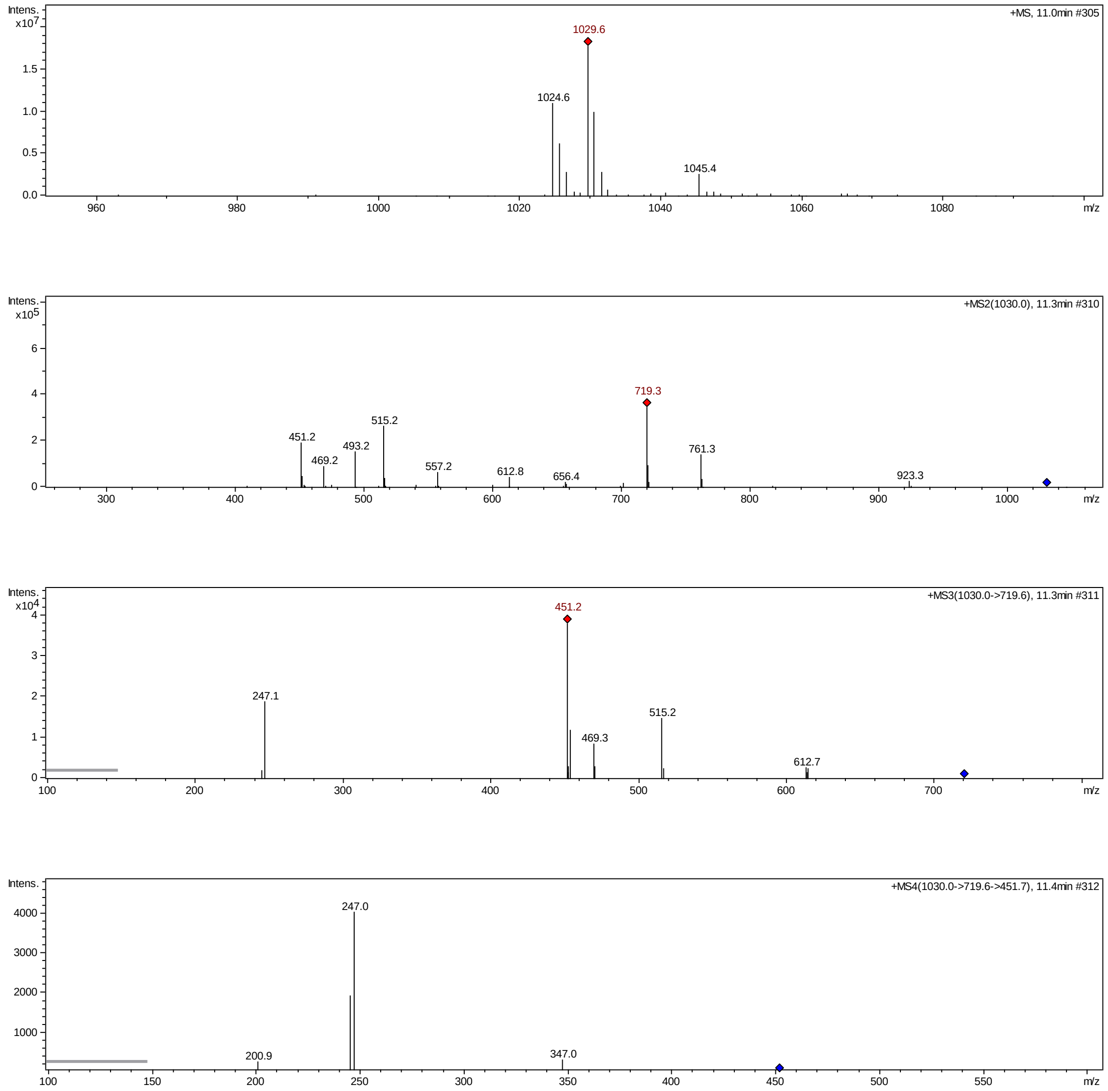


Figure S46. HPLC-ESI-MS$^n$ spectrum of peak **B5**.

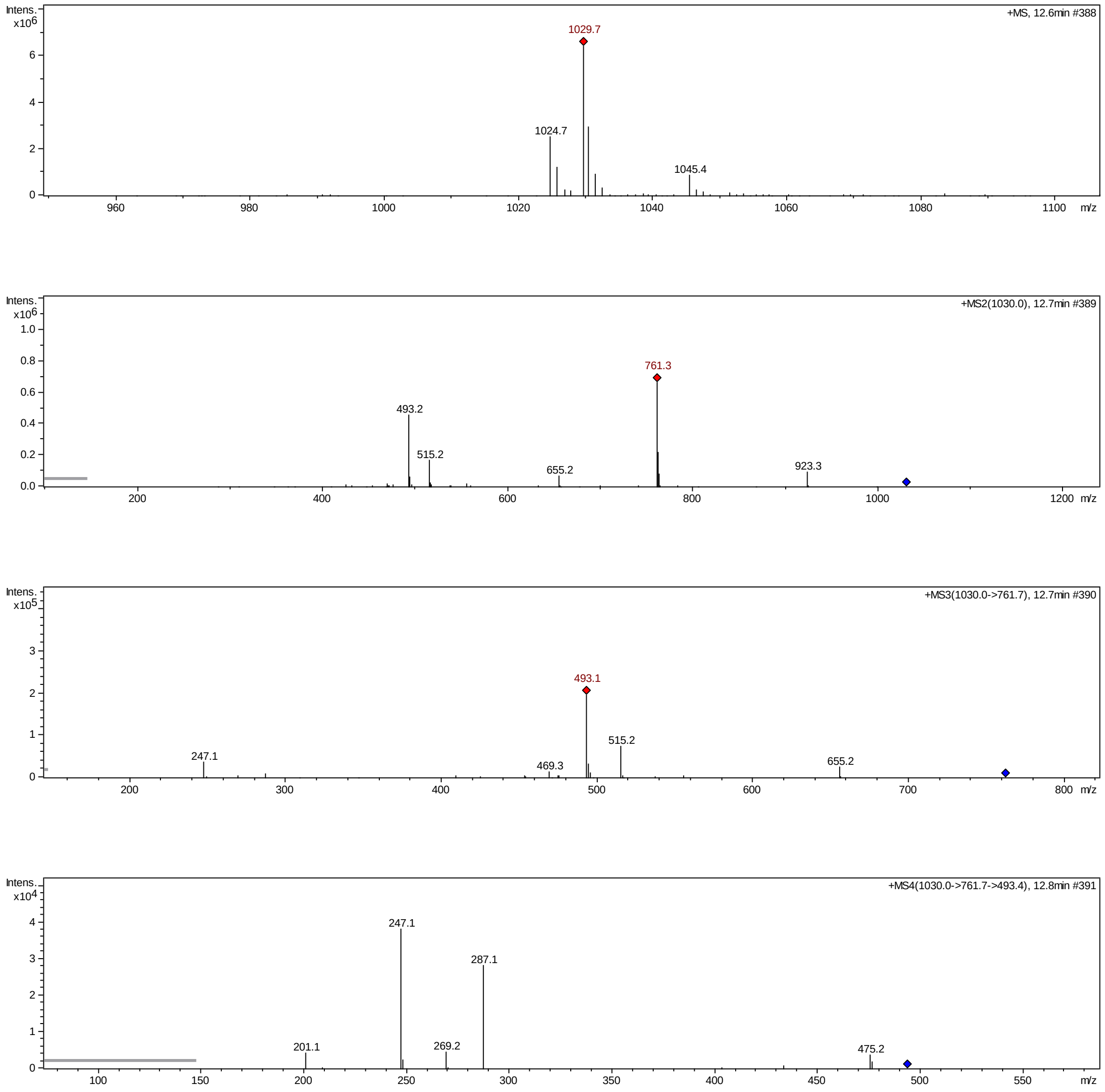


Figure S47. HPLC-ESI-MS$^n$ spectrum of peak **B6**.

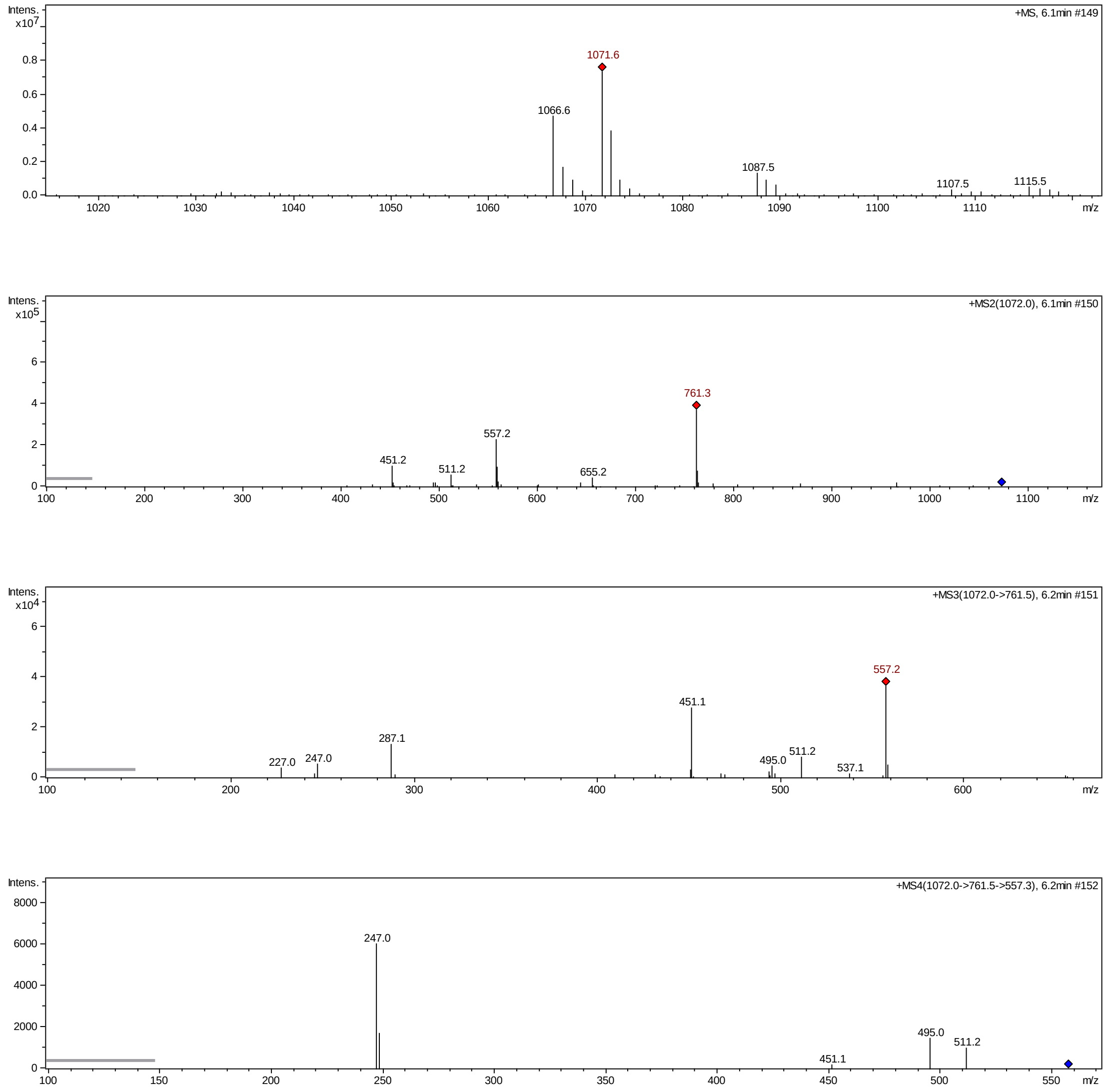


Figure S48. HPLC-ESI-MS$^n$ spectrum of Peak C1

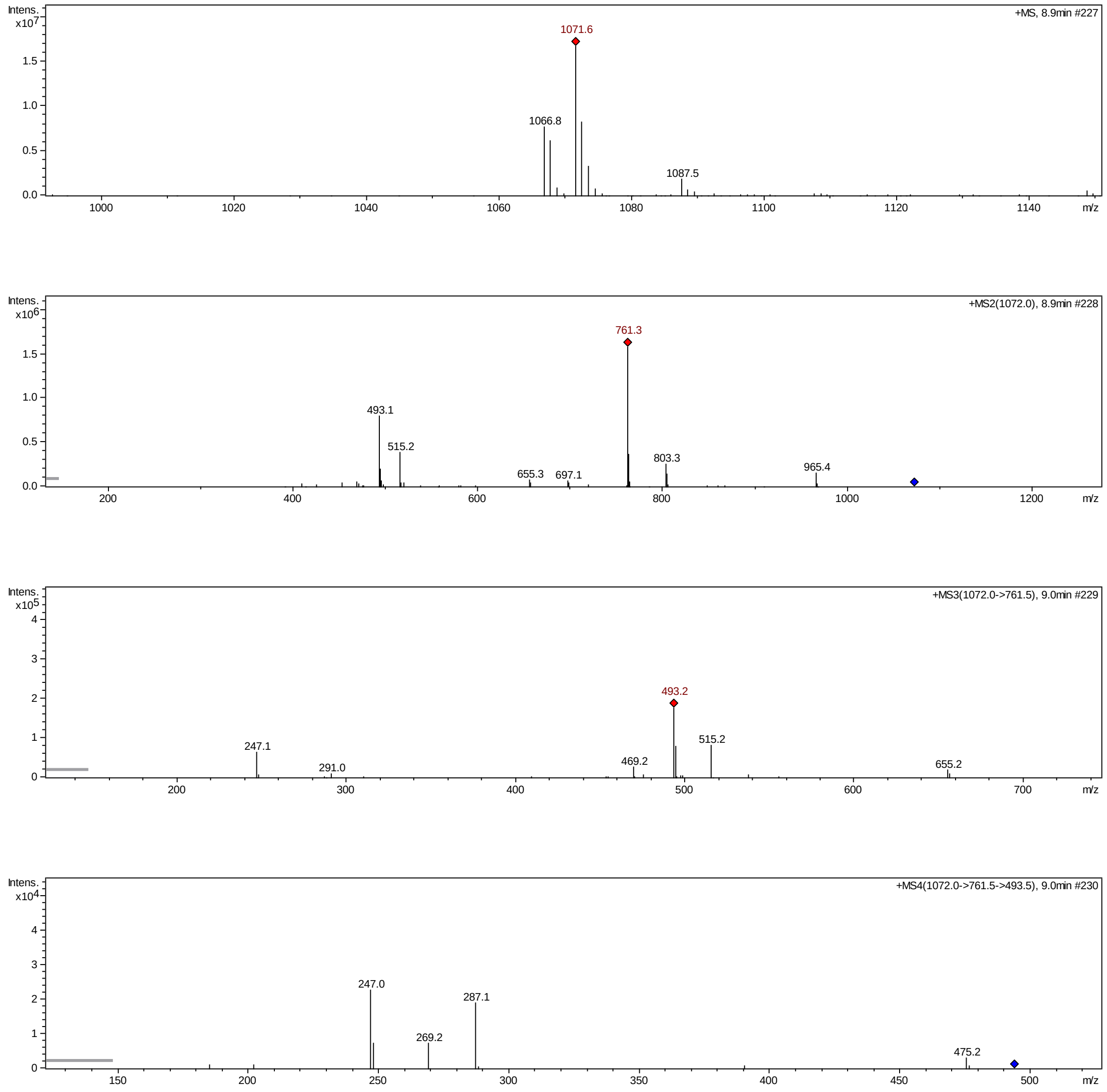


Figure S49. HPLC-ESI-MS$^n$ spectrum of Peak C2

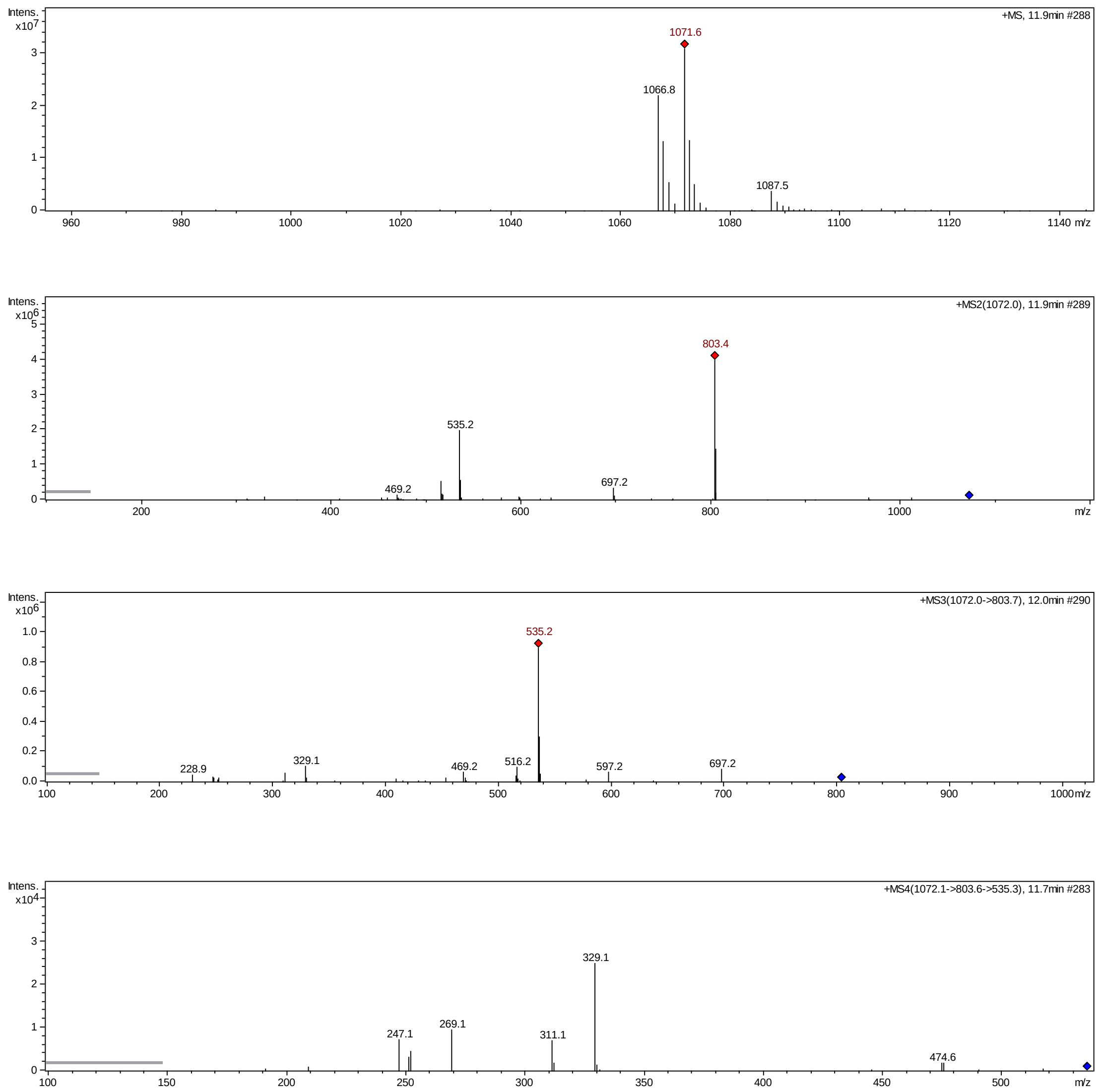


Figure S50. HPLC-ESI-MS$^n$ spectrum of Peak C3

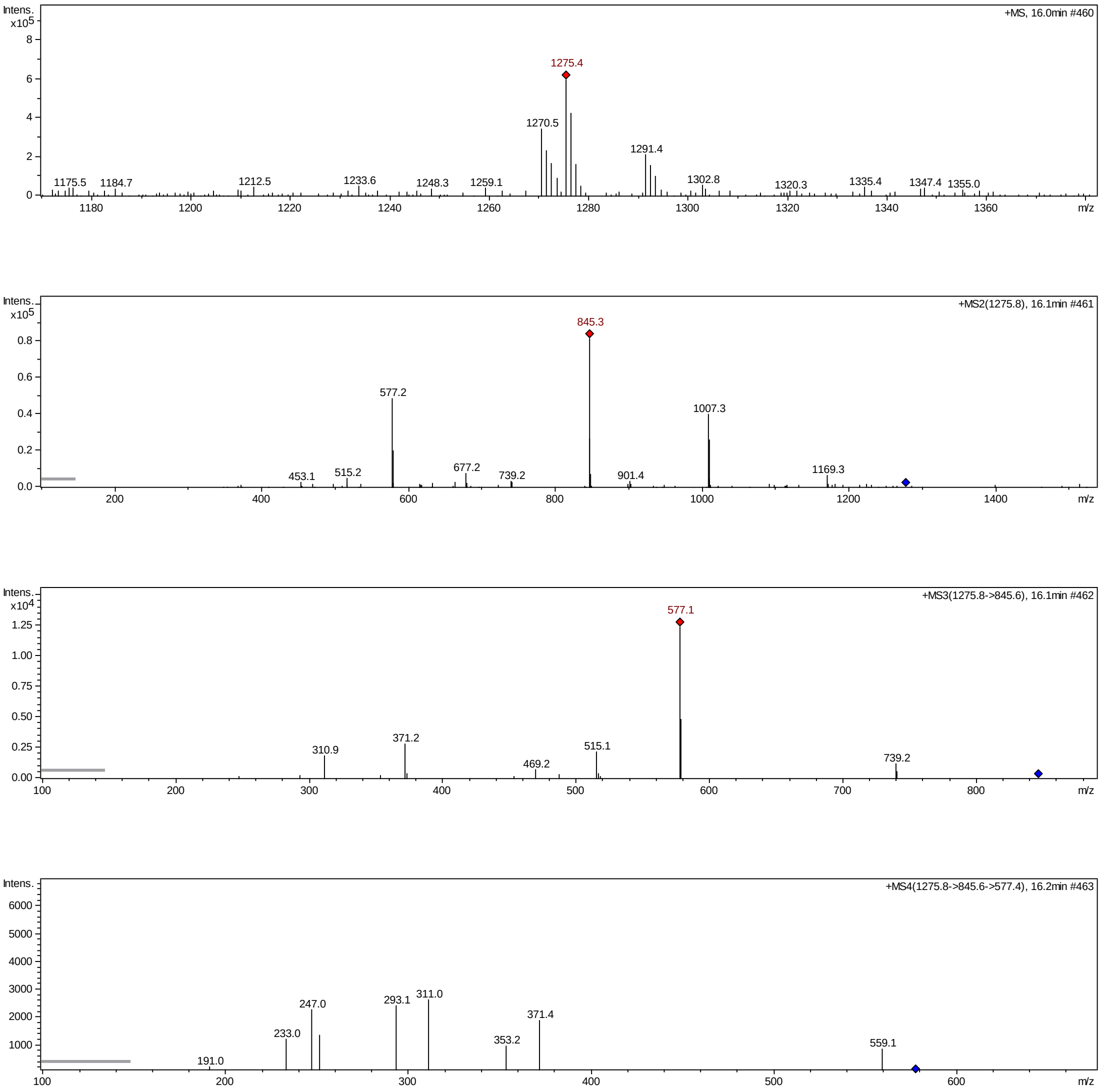


Figure S51. HPLC-ESI-MS$^n$ spectrum of peak **D1**.

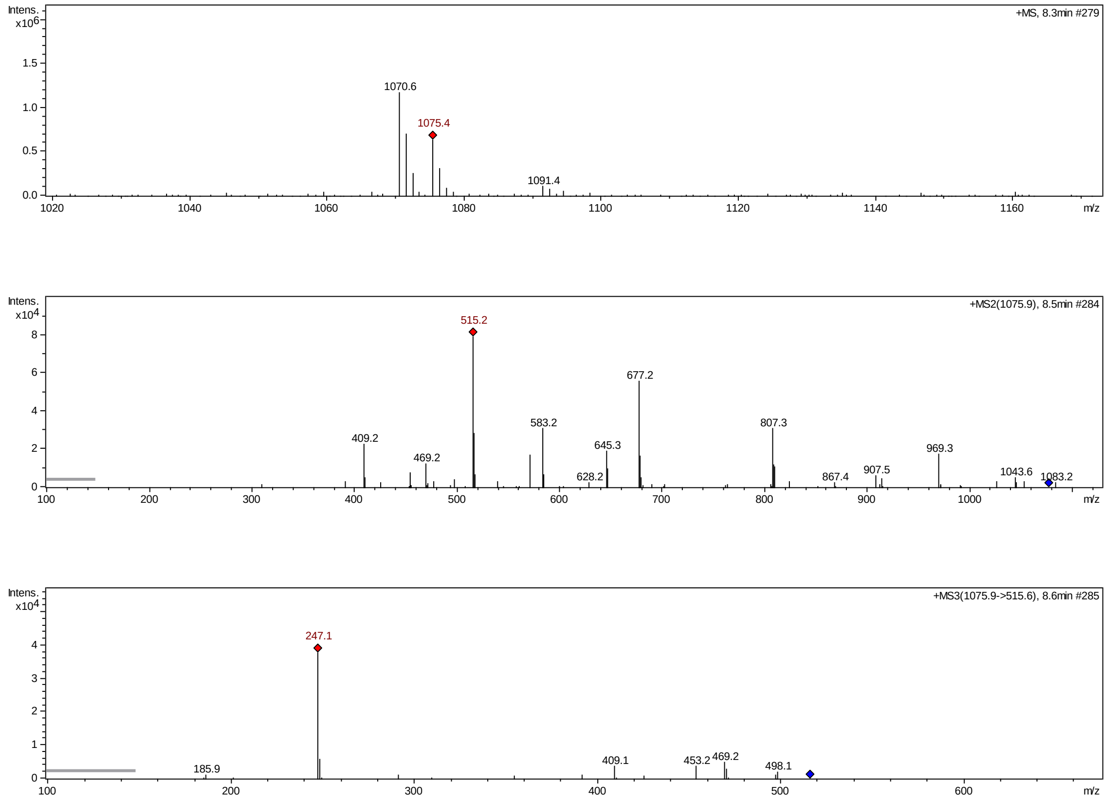


Figure S52. HPLC-ESI-MS$^n$ spectrum of peak **D2**.

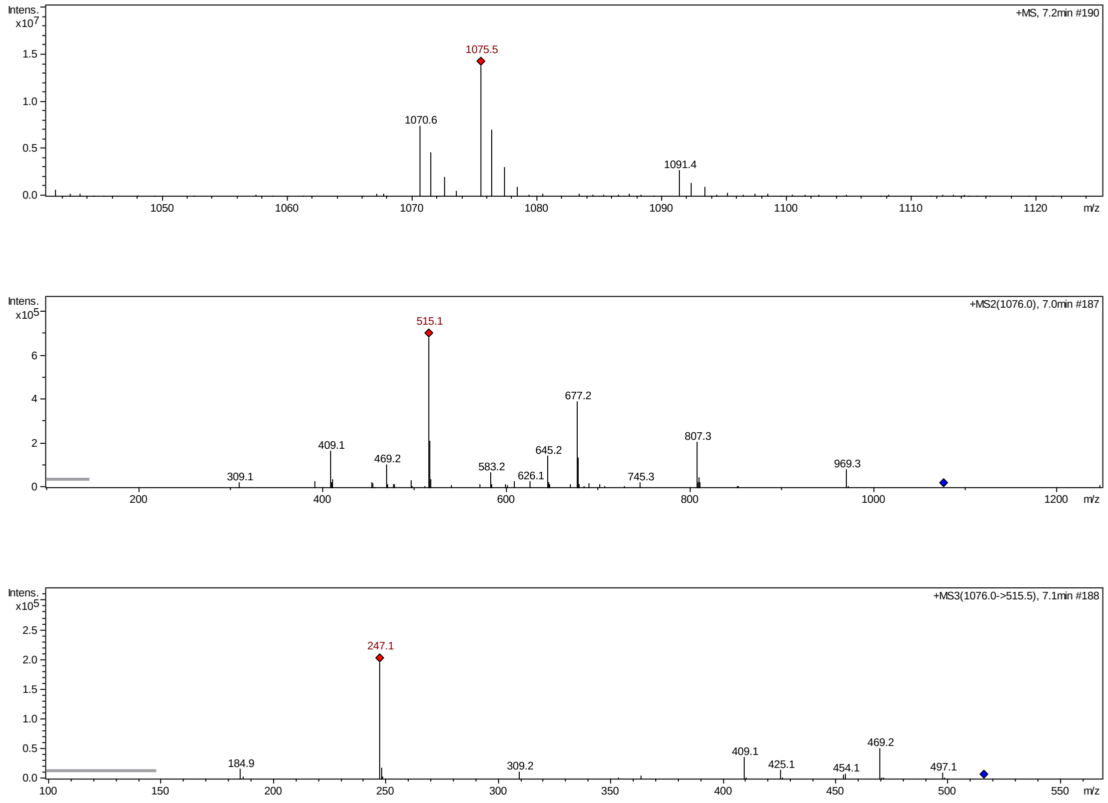


Figure S53. HPLC-ESI-MS$^n$ spectrum of peak **D3**.

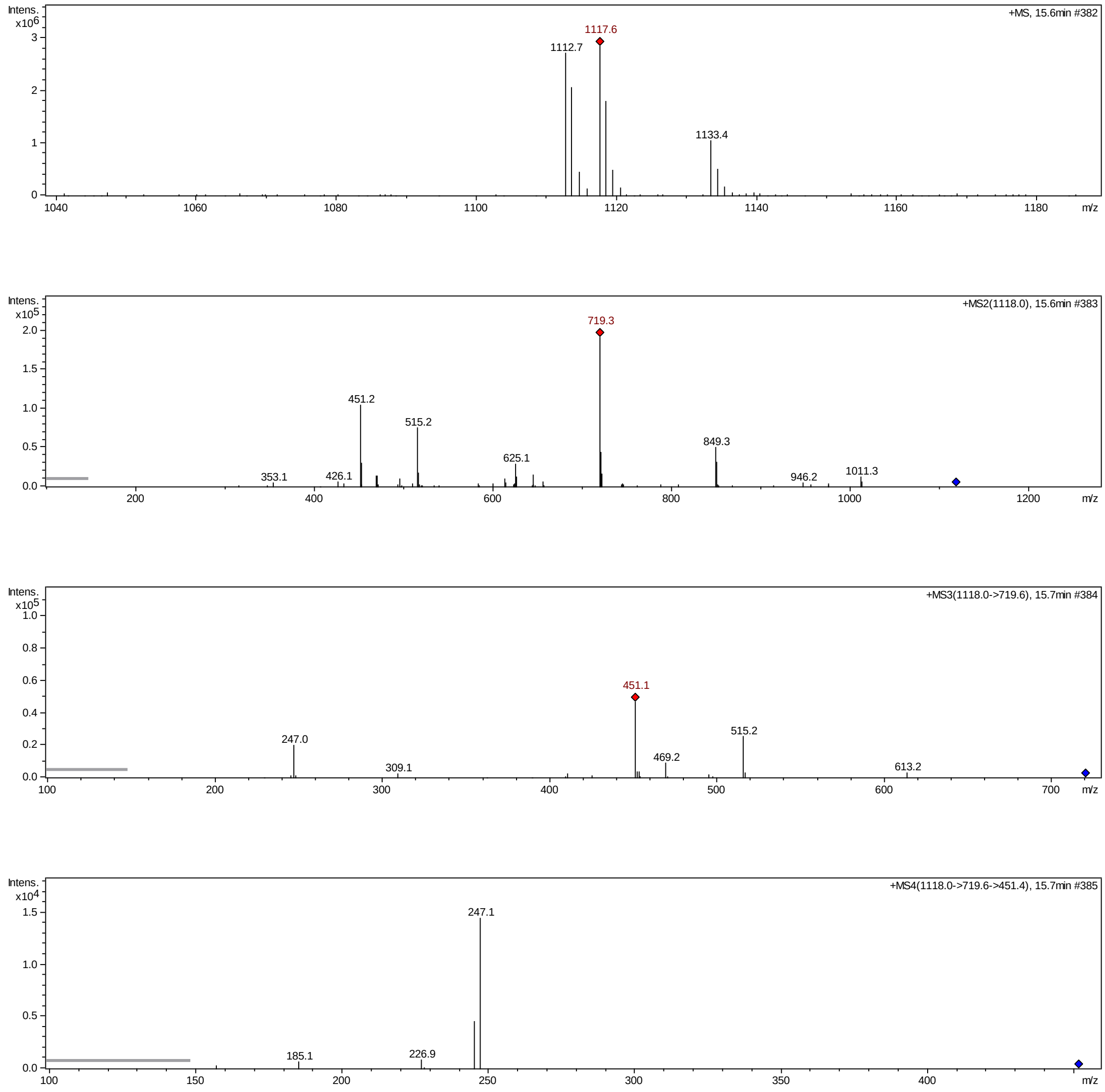


Figure S54. HPLC-ESI-MS$^n$ spectrum of peak **D4**.

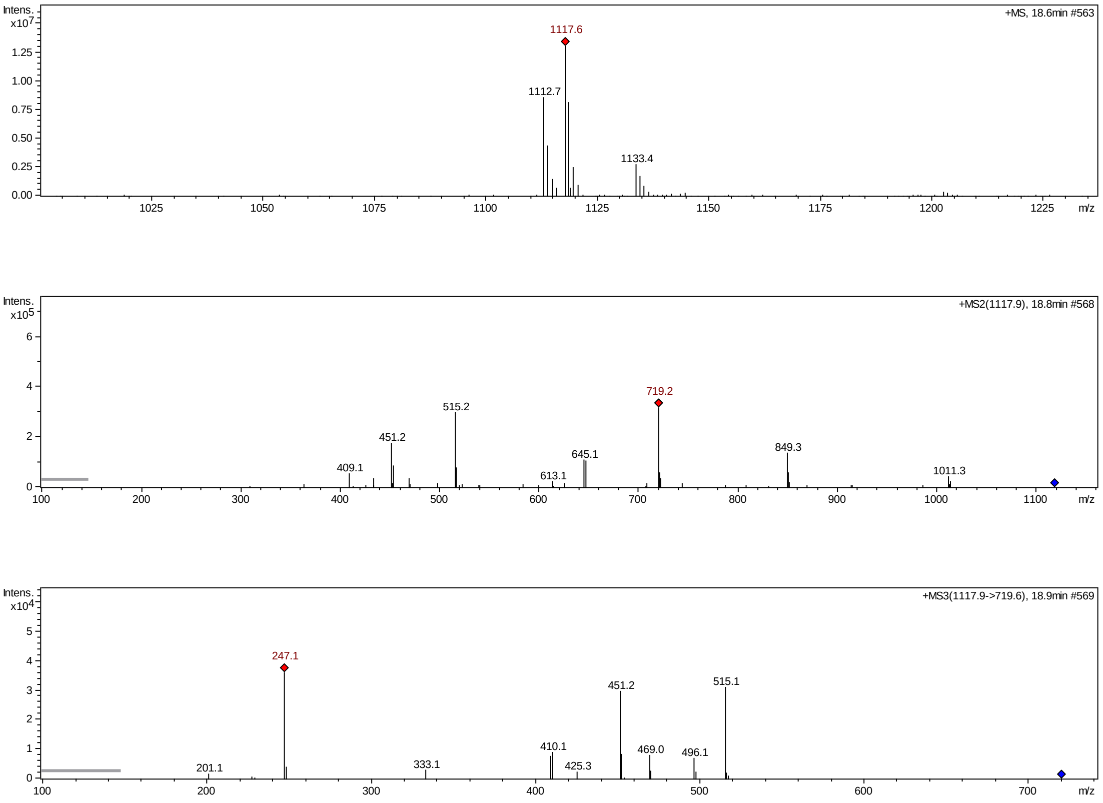


Figure S55. HPLC-ESI-MS$^n$ spectrum of peak **D5**.

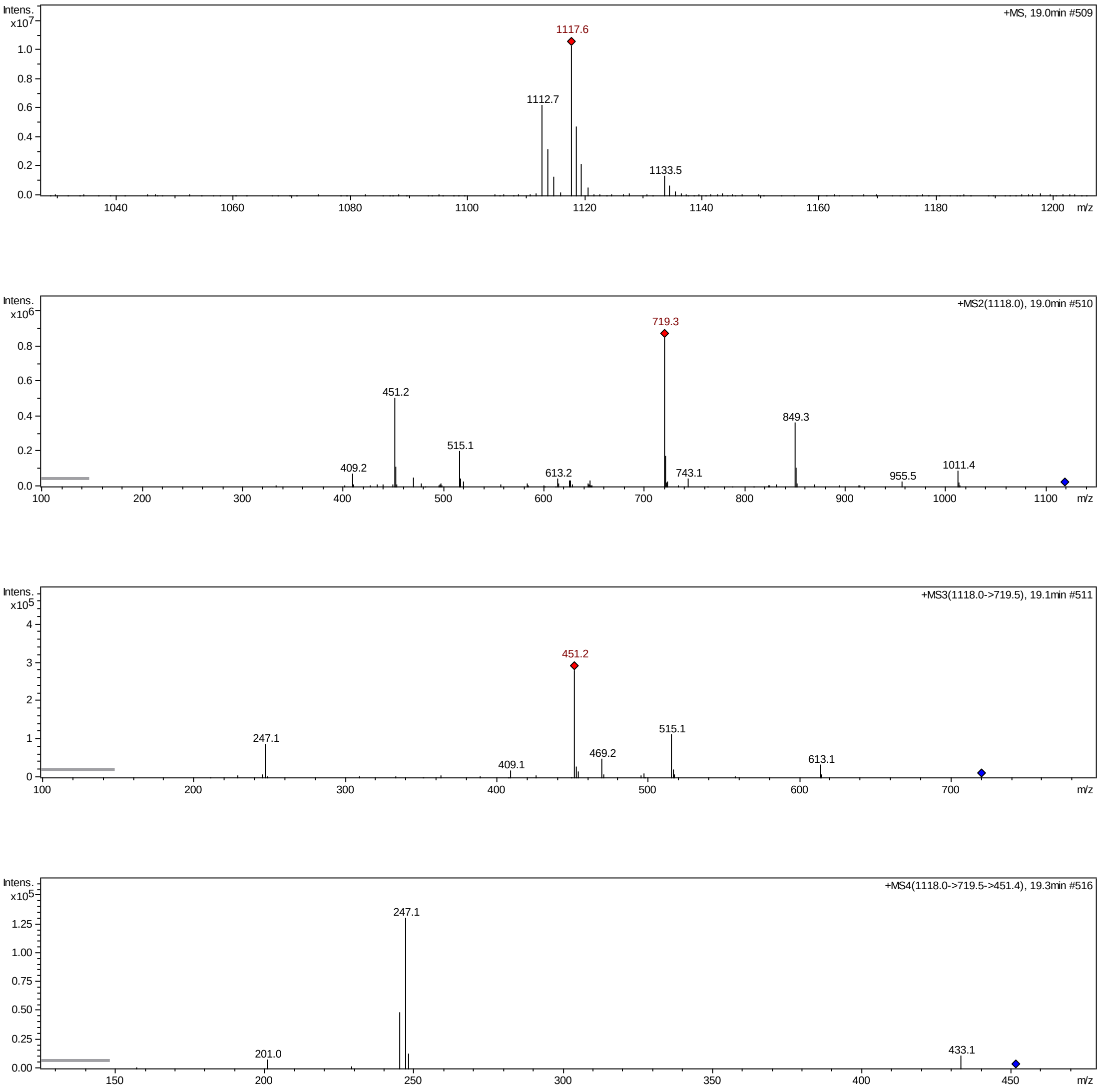


Figure S56. HPLC-ESI-MS$^n$ spectrum of peak **D6**.
